# Microdomain-Specific Modulation of L-Type Calcium Channels Leads to Triggered Ventricular Arrhythmia in Heart Failure

**DOI:** 10.1161/CIRCRESAHA.116.308698

**Published:** 2016-09-29

**Authors:** Jose L. Sanchez-Alonso, Anamika Bhargava, Thomas O’Hara, Alexey V. Glukhov, Sophie Schobesberger, Navneet Bhogal, Markus B. Sikkel, Catherine Mansfield, Yuri E. Korchev, Alexander R. Lyon, Prakash P. Punjabi, Viacheslav O. Nikolaev, Natalia A. Trayanova, Julia Gorelik

**Affiliations:** From the Department of Cardiovascular Sciences, Imperial Centre for Translational and Experimental Medicine, National Heart and Lung Institute (J.L.S.-A., A.B., A.V.G., S.S., N.B., M.B.S., C.M., A.R.L., P.P.P., J.G.), Department of Medicine (Y.E.K.), and Department of Cardiothoracic Surgery, Hammersmith Hospital, National Heart and Lung Institute (P.P.P.), Imperial College London, United Kingdom; Department of Biomedical Engineering and Institute for Computational Medicine, Johns Hopkins University, Baltimore, MD (T.O., N.A.T.); NIHR Cardiovascular Biomedical Research Unit, Royal Brompton Hospital, London, United Kingdom (A.R.L.); Institute of Experimental Cardiovascular Research, University Medical Center Hamburg-Eppendorf, Hamburg, Germany (S.S., V.O.N.); and Department of Biotechnology, Indian Institute of Technology Hyderabad, Kandi, Telangana, India (A.B.).

**Keywords:** electrophysiology, heart failure, L-type calcium channel, membrane microdomains, myocardial infarction

## Abstract

**Rationale:**

Disruption in subcellular targeting of Ca^2+^ signaling complexes secondary to changes in cardiac myocyte structure may contribute to the pathophysiology of a variety of cardiac diseases, including heart failure (HF) and certain arrhythmias.

**Objective:**

To explore microdomain-targeted remodeling of ventricular L-type Ca^2+^ channels (LTCCs) in HF.

**Methods and Results:**

Super-resolution scanning patch-clamp, confocal and fluorescence microscopy were used to explore the distribution of single LTCCs in different membrane microdomains of nonfailing and failing human and rat ventricular myocytes. Disruption of membrane structure in both species led to the redistribution of functional LTCCs from their canonical location in transversal tubules (T-tubules) to the non-native crest of the sarcolemma, where their open probability was dramatically increased (0.034±0.011 versus 0.154±0.027, *P*<0.001). High open probability was linked to enhance calcium–calmodulin kinase II–mediated phosphorylation in non-native microdomains and resulted in an elevated *I*_Ca,L_ window current, which contributed to the development of early afterdepolarizations. A novel model of LTCC function in HF was developed; after its validation with experimental data, the model was used to ascertain how HF-induced T-tubule loss led to altered LTCC function and early afterdepolarizations. The HF myocyte model was then implemented in a 3-dimensional left ventricle model, demonstrating that such early afterdepolarizations can propagate and initiate reentrant arrhythmias.

**Conclusions:**

Microdomain-targeted remodeling of LTCC properties is an important event in pathways that may contribute to ventricular arrhythmogenesis in the settings of HF-associated remodeling. This extends beyond the classical concept of electric remodeling in HF and adds a new dimension to cardiovascular disease.

## Abstract 2

Supplemental Digital Content is available in the text.

Heart failure (HF) is a major cause of morbidity and mortality, contributing significantly to global health expenditure. Sudden death due to arrhythmia is responsible for >50% of deaths among patients with HF and therefore preventing arrhythmia and ameliorating the risk of sudden cardiac death secondary to HF is a paramount clinical need.^1,2^ Pathophysiological remodeling of cardiac function in HF occurs at multiple levels and includes the alterations in ion channel profile, Ca^2+^-handling proteins, and proteins mediating cell–cell coupling, predisposing to arrhythmias.^3–7^ Recent studies have demonstrated that disruption of proper cellular organization accompanied by a progressive loss of transverse tubule (T-tubule [TT]) microdomains in HF may also have an impact on calcium cycling, thus, promoting the development of arrhythmogenic triggers.^8^ Specifically, it has been hypothesized^9,10^ that the communication between L-type Ca^2+^ channels (LTCCs) and ryanodine receptors (RyRs) is impaired in HF, perhaps indicating that LTCCs are less strictly confined to TTs. In addition, LTCC protein phosphorylation defects have been identified in HF.^3,11^ Therefore, we hypothesized that there is altered distribution of single LTCCs in cellular microdomains in HF after TT degradation, which results in channel dysfunction critically contributing to the development of arrhythmogenic triggers.

Here, we used the super-resolution scanning patch-clamp technique^12^ to probe the microdomain-specific localization of functional LTCCs with nanospatial resolution in normal and failing ventricular cardiac myocytes. We showed, in failing cells, dislocation of functional LTCCs to the sarcolemma surface (or crest membrane, a term based on micrograph topography in contrast to TT), where they are rarely present in healthy cardiac myocytes. Obtaining evidence from multiple imaging modalities, electrophysiology and biochemistry, we discovered that these relocated channels exhibit higher open probability (P_o_) and phosphorylation status, which we found to be linked to enhanced activity of calcium–calmodulin kinase II (CaMKII). The experiments were complemented by the development and use of a novel accurate HF computational model, which also includes single-channel behavior to ascertain that LTCC relocation to the cell crest combined with enhanced CaMKII activity gives rise to the measured P_o_ values. The model then demonstrated how this abnormal behavior leads to cell-level oscillations in membrane voltage and development of arrhythmogenic triggers, and how these propagate to become arrhythmias at the organ level. The combined experimental/simulation approach presented here provides a comprehensive understanding of how disease-induced remodeling at the microdomain level is manifested into dysfunction at the organ level.

## Methods

For full details of Methods section, please see the Online Data Supplement.

### Study Approval

All animal experiments were carried out in accordance with the United Kingdom Home Office Animals (Scientific Procedures) Act 1986 Amendment Regulations 2012, incorporating the EU Directive 2010/63/EU, which conforms to the Guide for the Care and Use of Laboratory Animals published by the US National Institutes of Health (NIH publication No. 85-23, revised 1996). Experiments on isolated human cardiac myocytes were approved by the Imperial College Institutional Review Board.

### Myocytes Isolation and TT Characterization

Failing ventricular myocytes were isolated from a 16 weeks post myocardial infarction rat model of HF (Online Figure I) and transplanted human hearts from patients with dilated cardiomyopathy (Online Table I). Age-matched sham-operated rats and human tissue biopsy samples (Online Table II) were used to isolate control, nonfailing cardiac myocytes. The subcellular TT system was visualized by confocal imaging of Di-8-ANEPPS–stained cells.^6^ Surface topography was characterized by scanning ion conductance microscopy, which uses a glass nanopipette as sensitive probe as described elsewhere.^13^

### Super-Resolution Scanning Patch-Clamp With Pipette Clipping Modification

After generating a topographical image of the cell surface by scanning ion conductance microscopy, the tip diameter of the pipette was widened by clipping^13^ to increase the area of attachment. The pipette was then lowered to a specific location until it touched the membrane and a high resistance seal was established. Single LTCC recordings were then performed in a cell-attached mode.^12^ Controlled widening of the scanning nanopipette tip is described in detail in the Online Data Supplement (Online Figure II and Online Figure III).

### Optical Mapping of Calcium Activity

Optical mapping of cells loaded with the Ca^2+^-sensitive fluorescent dye Fluo-4AM via CMOS camera ULTIMA-L (SciMedia, USA Ltd, CA; 1000 fps, 1.5–2 μm/pixel) was used to monitor localized changes in [Ca^2+^]_*i*_.^14^

### Western Blot

Western blotting was done using monoclonal anti-phospho-CaMKII (Thermo Scientific, MA1-047) α-tubulin (Sigma, T9026), and GAPDH (Santa Cruz Biotechnology, FL-335) primary antibodies, followed by secondary anti-mouse antibodies Amersham ECL detection (GE Healthcare). Western blots were analyzed by using ImageJ software.

### Statistical Analysis

All graphs and statistical analysis were performed using either GraphPad prism 5 or Origin version 6.1. Normality was tested using the Kolmogorov–Smirnov test. In cases where data failed the normality test, the nonparametric Mann–Whitney test was used instead of the unpaired Student *t* test. Statistical differences were assessed with Student *t* test, Mann–Whitney test, Kruskal–Wallis test, and Fisher exact test as appropriate. All data are expressed as mean±SEM. A value of *P*<0.05 was considered statistically significant.

### Computational Simulations

#### Single-Channel Human L-Type Ca^2+^ Current (*I*_Ca,L_)

Human ventricular cell electrophysiological behavior was represented by the O’Hara-Rudy model.^15^ To model stochastic single-channel behavior and determine the channel P_o_ for comparison with human experimental data, for *I*_Ca,L_, we used its Markov-equivalent representation (Online Figure IV). To evolve the channel gating in response to a 1-s voltage change step from resting state to −6.7 mV, we used the Gillespie Exact Algorithm. Barium simulations matched barium experiments; with this as validation, we extrapolated to physiological calcium simulations. Example single-channel sweeps are shown in Online Figure V.

Once single-channel current simulation results were generated and validated with experimental P_o_ measurements, the *I*_Ca,L_ model was reverted back to a Hodgkin–Huxley formulation, retaining the CaMKII mode definitions and the behavior of the equivalent Markov version. Failing crest LTCCs were assumed to operate in CaMKII-phosphorylated mode, based on experimental findings (see Results section of this article); their inactivation was via the slow-gating mode. Failing LTCCs in TTs and control LTCCs were sensitive to standard CaMKII, and so inactivation was both fast and slow. Ensemble current computed by summation of single-channel sweeps matched the deterministic Hodgkin–Huxley current (Online Figure VI).

### Models of Human Control and Failing Myocytes

The original O’Hara-Rudy myocyte model did not include a subsarcolemmal volume and, thus, did not allow for Ca^2+^ accumulation near the intracellular mouth of Crest LTCCs. We, thus, incorporated subsarcolemmal volume and related fluxes in the O’Hara-Rudy myocyte model based on the work of Grandi et al^16^ and Shannon et al.^17^ Description of how this was done can be found in the Online Data Supplement.

The original O’Hara-Rudy model included LTCCs exclusively at TT sites with dyadic intracellular face. Here, LTCCs in TTs sensed and contributed to dyadic Ca^2+^; channels newly added to the crest sensed and contributed to subsarcolemmal Ca^2+^. In each of TT and crest locations, LTCC permeability, PCa, representing whole-cell current density in TTs (PCa_TT_) and crest (PCa_Crest_) needed to be defined. We utilized experimental data obtained in this study to assign values to PCa_TT_ and PCa_Crest_ in control and failing human cells. The data used were % occurrence of LTCCs, LTCC single-channel current amplitude, and in failing cells, the degree of TT loss. Specific values for PCa_TT_ and PCa_Crest_ in control and failing human cells and the methodology by which they were assigned can be found in the Online Data Supplement.

Outside of *I*_Ca,L_ several factors were included to recreate an accurate HF model. Descriptions of the formulations for Na^+^/Ca^2+^ exchanger, representation of orphaned RyRs, and additional parameters representing others HF ion channel remodeling in the myocyte model can be also be found in the Online Data Supplement.

### Whole Heart Model

Organ-level simulations were performed using an MRI-based, anatomically realistic human left ventricular model as described previously.^18^ Fiber orientation was assigned using a rule-based approach.^19^ A computational mesh at a resolution of <300 μm was generated using a validated approach.^20^ Transmural cell types and conductivity were specified according to experimental data from human left ventricles^7^ (Online Figure VII). All parameters defining differences between the endocardial and epicardial cell variations of the O’Hara-Rudy model were scaled linearly across the ventricular walls to generate smoothly varying intermediate transmural types (10 segments defined using a Laplace-Dirichlet technique).^19^ Pacing was delivered to the apex using a 2-cm diameter electrode. Electrophysiological simulation and numeric methods were identical to previous work.^18^ Simulations were executed in monodomain mode using the CARP software package.^21,22^

## Results

HF is associated with the loss of TTs with consequent alterations in LTCC spatial distribution and their functional properties.

**Figure 1.**
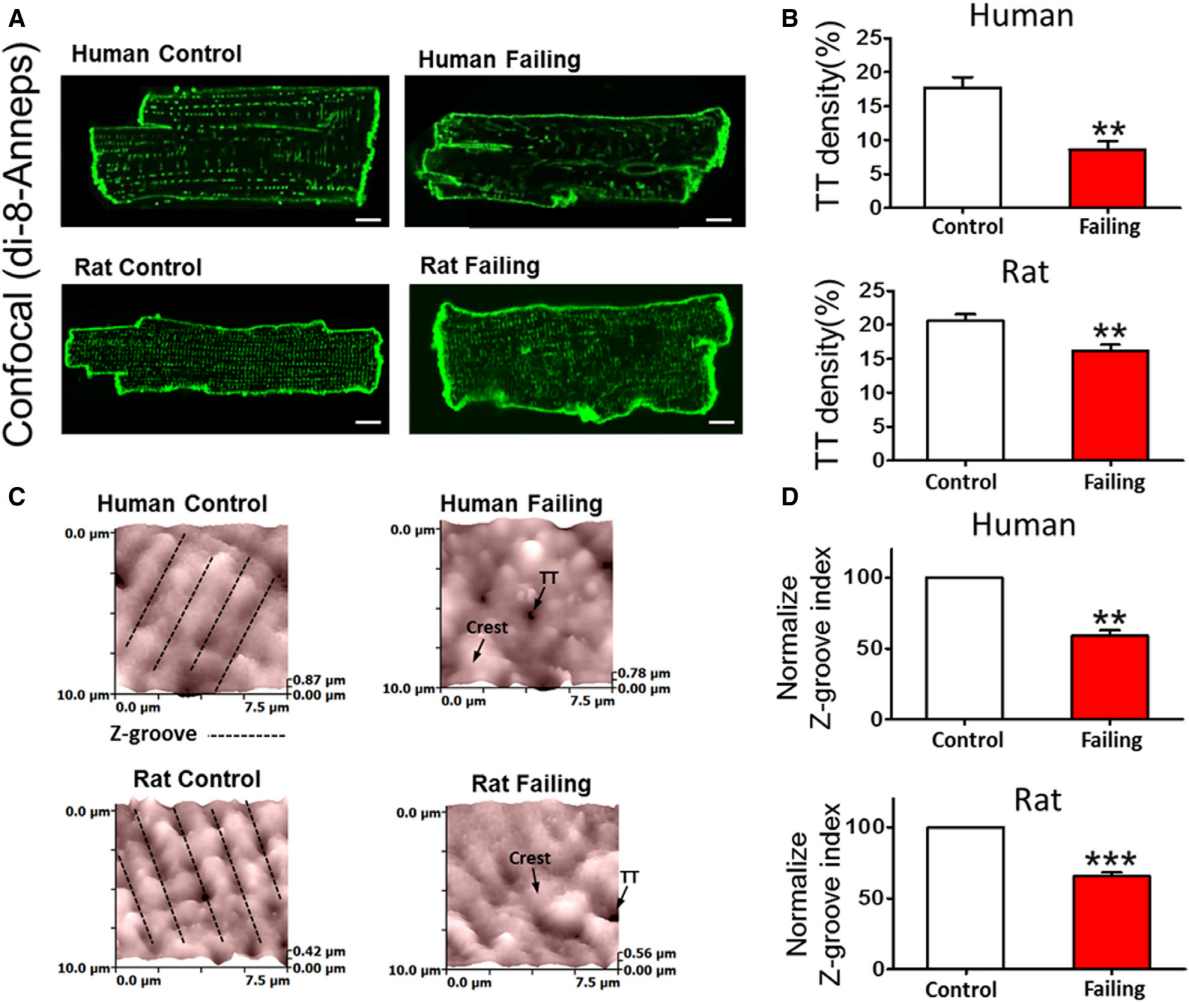
**T-tubule (TT) loss in ventricular myocytes from failing human and rat hearts. A**, Example confocal images of human control and failing (**top**) and rat control and failing (**bottom**) cardiac myocytes showing membranes stained with Di-8-ANEPPS; scale bar, 10 μm. **B**, TT density in control and failing cells (human control n=20, failing n=8; rat control n=20, failing n=20; **P<0.01 by unpaired Student *t* test). **C**, Example scanning ion conductance microscopy scans from a 10×10 μm portion of cell membrane shows regular undulations, indicating spatially alternating TT invaginations and surface membrane crests in human (**top left**) and rat (**bottom left**) control cardiac myocytes that are relatively absent in failing cells (**right**). **D**, Z-groove index in human and rat failing cells normalized to control average value (human control n=30, failing n=59; **top**, *P*<0.01; rat control n=91, failing n= 122; **bottom**, *P*<0.001 by unpaired Student *t* test).

In both rat and human failing myocytes, we observed a significant decrease in internal TT density when compared with nonfailing controls (Figure 1A and 1B; ≈50% decrease in human, *P*<0.01), consistent with our previous findings.^6^ Using scanning ion conductance microscopy,^23^ we found that the loss of TT correlates with the loss of surface structures as well (Figure 1C). Using Z-groove index as metric of surface integrity,^24^ we found that in both human and rat failing myocytes, surface structure was impaired (Figure 1D, ≈40% reduction in human, *P*<0.01). We also detected reduction in the number of TT openings on the surface of failing cardiac myocytes, identified as dark circles in scanning ion conductance microscopy images (≈35% reduction in rat compared with control, *P*<0.001; Online Figure VIII).

**Figure 2.**
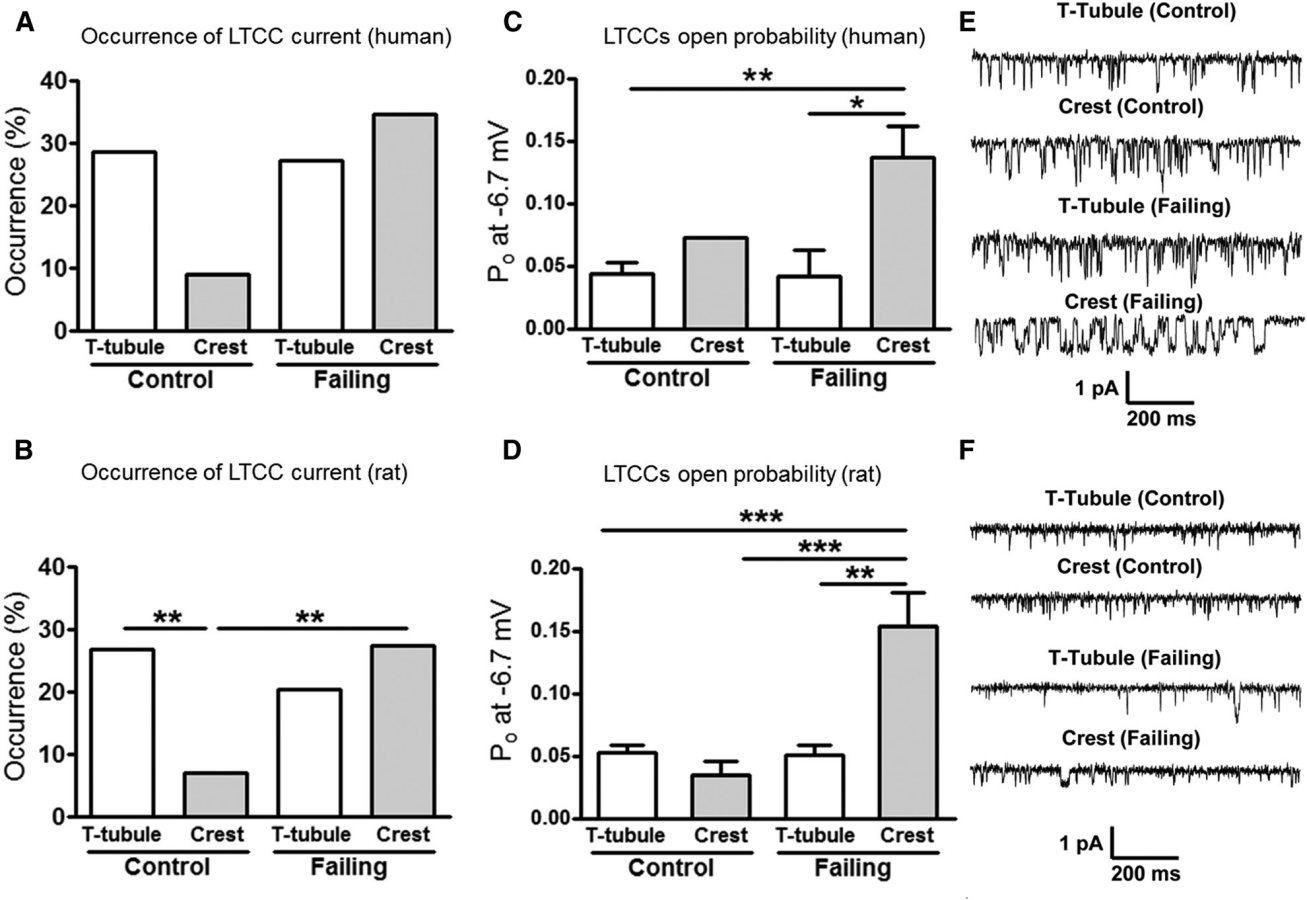
**Abnormal L-type Ca^2+^ channel (LTCC) localization and function in failing cardiac myocytes.** Chance of obtaining an LTCC current (% occurrence) in human (**A**) and rat (**B**) control and failing cells (**P<0.01 by Fisher exact test). Open probability (P_o_) during a maximum activating voltage step (to −6.7 mV) in human (**C**) and rat (**D**) failing cells (human control, T-tubule [TT] n=6, crest n=1; failing, TT n=6, crest n=9, * *P*<0.05, ** *P*<0.01 by Mann–Whitney test; rat control, TT n=19, crest n=4; failing, TT n=11, crest n=13, ** *P*<0.01, ***P<0.001 by Mann–Whitney test). Representative single-channel traces at −6.7 mV showing LTCC activity in human (**E**) and rat (**F**) cardiac myocytes.

The loss of TT microdomains in failing myocytes was accompanied by altered spatial distribution of LTCCs. In control rat and human cardiac myocytes, LTCC activity was predominantly recorded in TTs (26.7% of 86 successful patches in rat and 28.6% of 21 successful patches in human cardiac myocytes showed LTCC activity) as opposed to the crest, where LTCC activity was rarely recorded (only 7.02% of 57 successful patches in rat and 9.1% of 11 successful patches in human cells showed LTCC activity, Figure 2A and 2B, control). This confirmed our previous observation that the majority of functional LTCCs reside in the TTs.^12^ Interestingly, LTCC occurrence along the Z-groove in rat cardiac myocytes was found to be intermediate between that in TT and crest areas (15% of 20 successful patches), suggesting a density gradient of channels throughout the membrane.

In contrast, in failing rat cardiac myocytes, LTCC current was recorded with similar frequency from both TTs and crests (20.4% of 54 successful patches in the TTs versus 27.3% of 55 successful patches in the crest showed LTCC activity). In failing human cardiac myocytes, LTCC activity was even higher in the crest (27.3% of 22 successful patches in the TTs versus 34.6% of 26 successful patches in the crest showed LTCC activity). This indicates that the distribution of functional LTCCs in the cardiac myocyte membrane was significantly altered in HF (Figure 2A and 2B, failing).

Our experiments demonstrated that the HF-associated increase in the number of functional LTCCs outside of their native microdomains is accompanied by the changes in their behavior. The LTCC P_o_ was significantly elevated at the crest of failing myocytes when compared with that in crest of control myocytes in rat (P_o_ at −6.7 mV: 0.034±0.011 for control crest LTCCs versus 0.153±0.026 for failing crest LTCCs, *P*<0.001; Figure 2D) and to that in TTs (0.053±0.005 for control TT LTCCs, *P*<0.001; 0.051±0.008 for failing TT LTCCs, *P*<0.01; Figure 2D and 2F). No changes in LTCC amplitude were observed in rat HF (Online Figure IX). In failing humans myocytes, the P_o_ was also significantly elevated at the crest when compared with that in TT of control and failing myocytes (P_o_ at −6.7 mV: 0.136±0.025 for failing crest LTCCs versus 0.043±0.01 for control TT LTCCs, *P*<0.01 and versus 0.042±0.021 for failing TT LTCCs, *P*<0.05; Figure 2C and 2E). We next endeavored to determine the mechanisms for LTCC functional changes in HF.

### Constitutive Phosphorylation of LTCCs by Cytoplasmic CaMKII Leads to an Increase in LTCC P_o_

Elevated phosphorylation can lead to increased P_o_ of LTCCs.^25^ Also, CaMKII activity is elevated in HF,^26^ and CaMKII can phosphorylate LTCCs at specific sites.^27^ Therefore, we tested the hypothesis that the elevated activity of CaMKII in failing cardiac myocytes is responsible for phosphorylation and, thus, for the high P_o_ of LTCCs in crest.

**Figure 3.**
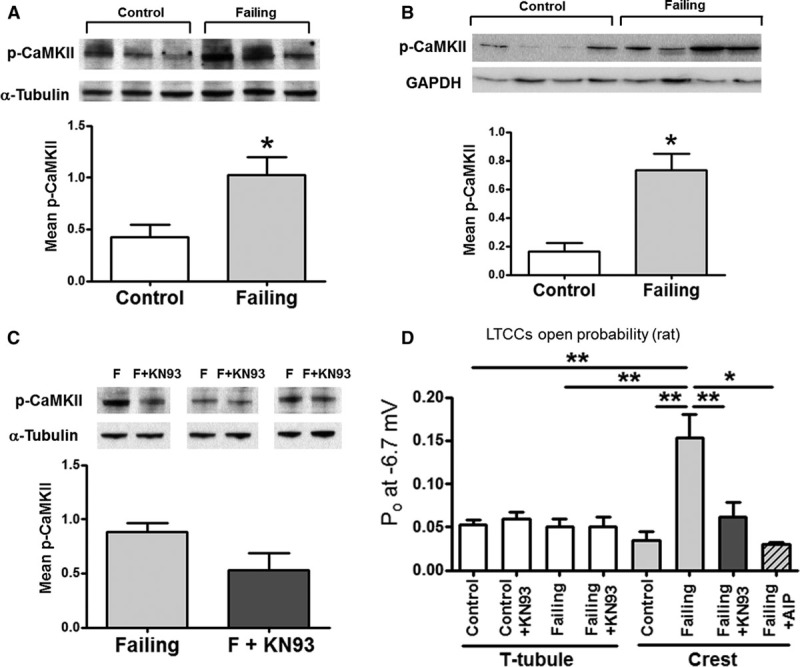
**L-type Ca^2+^ channels (LTCCs) at crests in rat failing myocytes are hyperphosphorylated by Ca^2+^ calmodulin-dependent protein kinase II (CaMKII). A**, Mean phosphorylated CaMKII (Thr286) from whole-cell lysate in control and failing cardiac myocytes. A representative blot is shown above the graph. Results are normalized to α-tubulin (**B**) Mean p-CaMKII from whole tissue from control and failing human samples. A representative blot is shown above the graph. Results are normalized to GAPDH. **C**, Mean p-CaMKII from whole-cell lysate in failing cardiac myocytes with or without CaMKII inhibition. A representative blot is shown above the graph. Results are normalized to α-tubulin. **D**, LTCC open probability (P_o_) in control and failing rat myocytes, under control conditions and after CaMKII inhibition with 5-μmol/L KN-93 or with 5-μmol/L autocamptide-2 related inhibitor peptide (AIP; control T-tubule [TT] n=19; control TT+KN-93 n=5; failing TT n=11; failing TT+KN-93 n=4; control crest n=4; failing crest n=13; failing crest+KN-93 n=9; failing crest+AIP n=3. * *P*<0.05, **P<0.01 by Kruskal–Wallis test).

Western blots indicated that the phosphorylated CaMKII T286 was higher in rat and human failing cells (Figure 3A and 3B); this p-CaMKII could be reduced after the application of the CaMKII inhibitor KN-93 (Figure 3C; only rat myocytes were used in these experiments because of the paucity of human cells). Because this residue is critical for the association and phosphorylation of the LTCC β_2a_ subunit,^28^ these data indicate that LTCC phosphorylation should be increased. Besides, as we have shown in Figure 3D and Online Figure X, local P_o_ measurements demonstrate that LTCC P_o_ in the crest of failing myocytes was reduced to a value similar to control after the application of KN-93 (from 0.153±0.026 for failing crest LTCCs to 0.061±0.018 for failing crest LTCCs with KN-93, *P*<0.01). LTCCs from control cells or from TT failing cells were not affected by the inhibitor (Figure 3D), suggesting that the increase of CaMKII in failing cells had only effect on the failing myocyte crest, confirming our hypothesis. It has been shown that KN-93 can block LTCC directly under certain conditions^29^; to strengthen our conclusion, autocamptide-2–related inhibitor peptide was used on failing crest channels (Figure 3B) and the results showed the reduction of P_o_ to control values (0.031±0.003, *P*<0.05 versus failing crest values), confirming our initial findings.

### Abnormal [Ca^2+^]_i_ Behavior in Failing Cells

**Figure 4.**
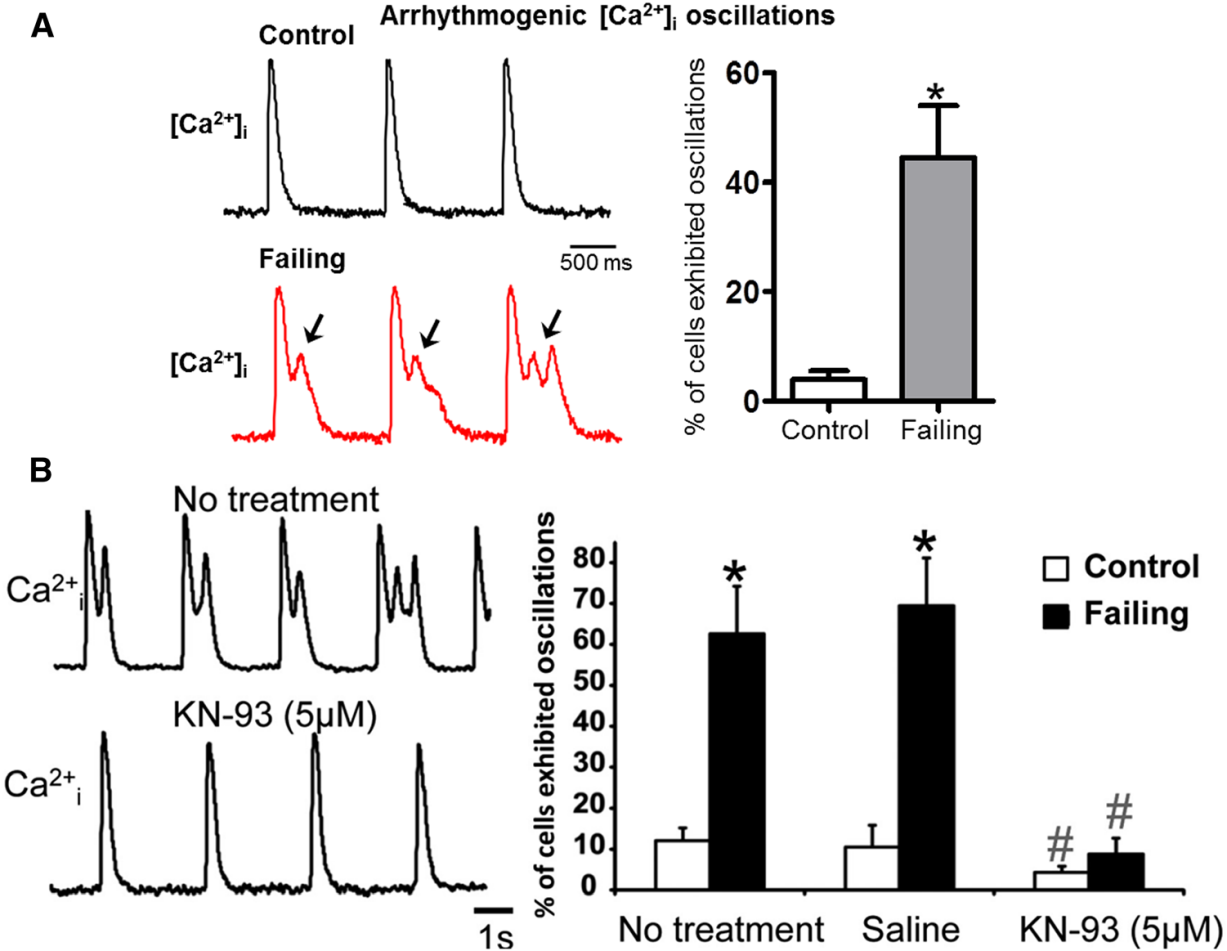
**Ca^2+^ oscillations that develop in failing but not in control rat cardiac myocytes are Ca^2+^ calmodulin-dependent protein kinase II dependent. A, Left**, Spontaneous [Ca^2+^]_i_ oscillation occurred at a pacing rate of 0.5 Hz during the decay phase of calcium transients in failing rat cardiac myocytes only. **Right**, Percentage of cells exhibiting Ca^2+^ oscillations (control n=106, failing=73, * *P*<0.05 by Mann–Whitney test). **B**, **Left**, Example of Ca^2+^ transients recorded from the same failing cardiac myocytes before and after treatment with KN-93. **Right**, Number of cells (in %) exhibiting Ca^2+^ oscillations (n=2 rats, 32–114 cardiac myocytes in each group; * *P*<0.05 for failing vs control; ^#^*P*<0.05 for KN93 effect within the same group by Mann–Whitney test). Saline mimics no treatment.

Because HF remodeling altered LTCC function in failing myocytes, we investigated whether there were concomitant changes in Ca^2+^ transients. Optical mapping of Ca^2+^ transients at pacing rates of 0.5 to 1 Hz revealed spontaneous [Ca^2+^]_i_ oscillations, which occurred during the decay phase in a greater proportion of failing cardiac myocytes compared with control (0.5 Hz, ≈45% failing versus ≈4% control *P*<0.05; Figure 4A). These Ca^2+^ oscillations were completely suppressed after treatment with KN-93 (Figure 4B) but not by KN-92 (Online Figure XI), an inactive analog of KN-93. This suggests that the phosphorylation inhibition by KN-93 reduces the late LTCC current resulting from the increased LTCC P_o_ at the cell crest and prevents [Ca^2+^]_i_ oscillations.

In rat failing cardiac myocytes, whole-cell patch-clamp recordings (Online Figure XII) show prolonged action potential (AP) duration similar to what had been previously described in HF.^30,31^ It has been shown that such AP duration prolongation is accompanied by spontaneous occurrence of single and multiple early afterdepolarizations (EADs) in failing ventricular myocytes.^31^ Oscillations in the Ca^2+^ transient such as shown in Figure 4, occurring in the settings of HF-associated ion channel remodeling, are also widely associated with EADs, a class of possible arrhythmogenic triggers in the heart.^32^ However, linking Ca^2+^ transient oscillations to the development of HF-related arrhythmias at the organ level is a challenging task, both in terms of demonstrating by experimentation, across the spatial scales of structural hierarchy, and also causally, as electrotonic influences at the tissue/organ level could suppress cell-level triggers.^33^ To prove that HF-induced changes in microdomain localization of LTCCs and their functional consequences at the cellular level could result in arrhythmia in the failing human heart, we developed and utilized a novel computational model of human HF that incorporated the experimental findings described above.

### Computational Model Based on Single-Channel Kinetics Predicts the Development of Cellular-Level Triggers of Arrhythmias in Human HF

**Figure 5.**
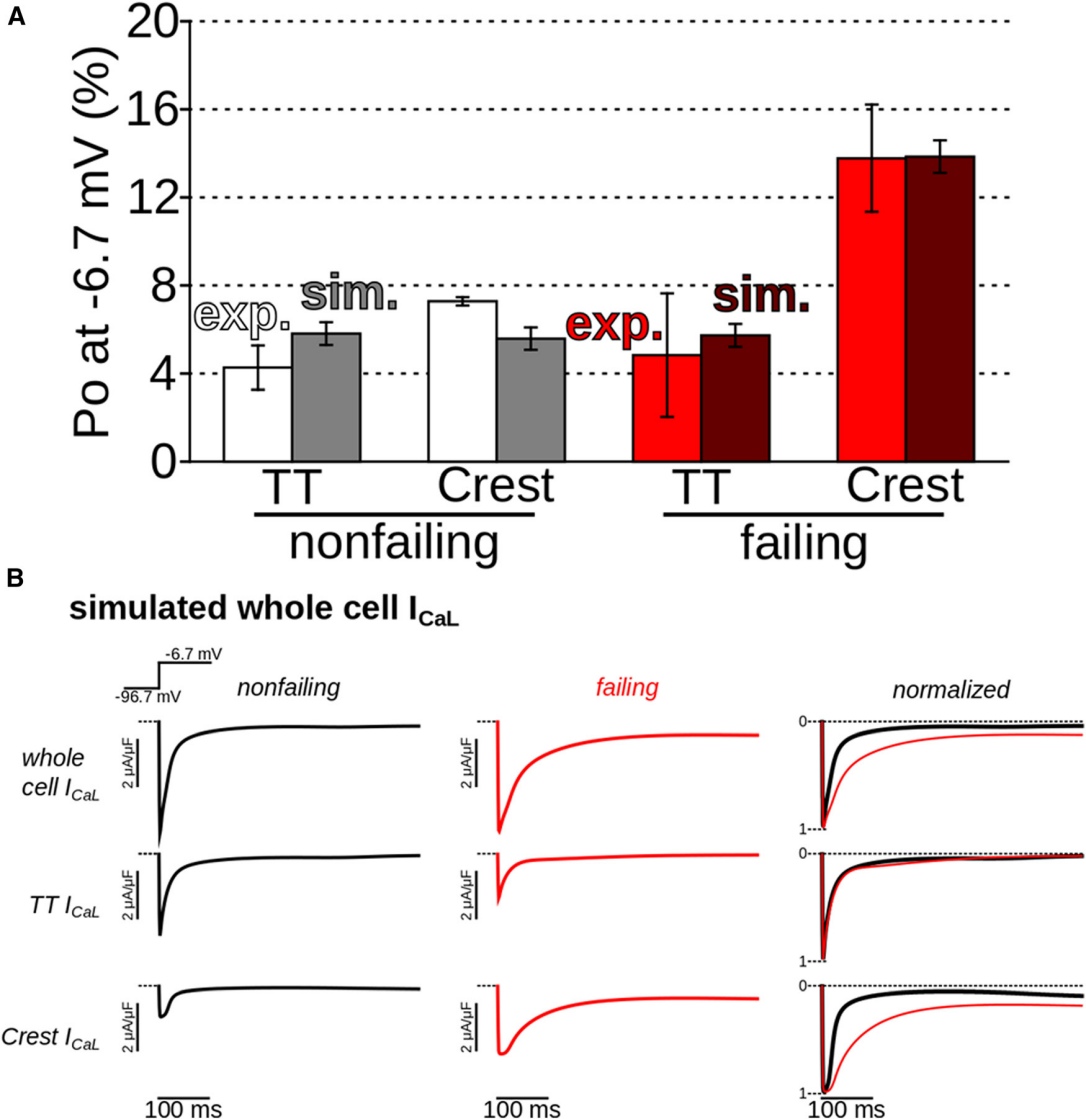
**Simulations demonstrated increased open probability (P_o_) of L-type Ca^2+^ channels (LTCCs) at failing crest sites, as in experiments, and slowed inactivation. A**, Comparison between simulated and experimental P_o_ values for 1-s voltage steps to −6.7 mV (average of 500 stochastic sweeps in simulations). **B**, Simulated whole-cell T-tubule (TT) and crest LTCC current (*I*_Ca,L_) in control (black) and failing (red) cells. Traces in right column were normalized to allow visual comparison of decay rates.

The human HF model was based on the Monte Carlo simulations of the kinetics of a single human LTCC (Online Data Supplement). Incorporating the experimental finding that in failing cardiac myocytes LTCCs at crests are CaMKII-phosphorylated, we calculated P_o_ values in TTs and crests in control and failing human myocytes (Figure 5A, example sweeps in Online Figure V). Simulations were able to relate LTCC phosphorylation at crests to the elevated local P_o_ value. The close match between simulation and experimental results served as a validation of the model, allowing us to use simulations to explore the downstream effect of LTCC functional changes.

Simulated human whole-cell Ca^2+^current is of similar magnitude in failing and control cells, consistent with previous findings^34–37^ and our results on failing rat cardiac myocytes (Online Figure XIII). Current decay is slower in failing myocytes compared with control (Figure 5B), suggesting a potential for destabilization of repolarization. Slower decay is because of enhanced CaMKII phosphorylation of LTCC channels in the crest. Traces corresponding to TTs were nearly identical for failing and control cells with regard to decay rate; however, TTs in failing myocytes were depopulated and thus current magnitude in TT was reduced relative to control.

**Figure 6.**
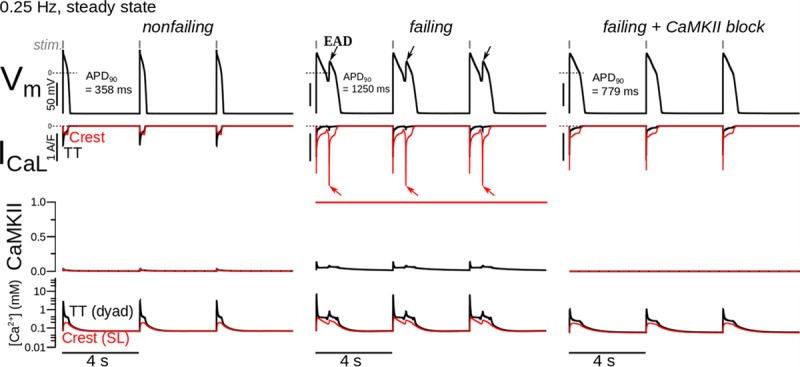
**Simulations demonstrating the development of proarrhythmic triggers (early afterdepolarizations [EADs]) in failing human ventricular (endocardial) myocytes.** Rows from top to bottom show simulated action potentials, T-tubule (TT, black) and crest (red) *I*_Ca,L_, TT and crest Ca^2+^ calmodulin-dependent protein kinase II (CaMKII) activity (0.0–none, no L-type Ca^2+^ channels [LTCCs] phosphorylated; 1.0–maximal, all LTCCs phosphorylated), and TT and crest Ca^2+^ concentrations (ie, concentrations in the dyadic and subsarcolemmal volumes). Columns from left to right show cases of control, failing, and failing cells with CaMKII block.

The LTCC kinetics model was incorporated, together with other HF electrophysiological alterations, in an AP model of the human (endocardial) ventricular myocyte to determine the cell-level consequences of LTCC dysfunction. AP simulation results showed that EADs developed because of late L-type Ca^2+^ current appearance in failing myocytes only (Figure 6). Here, CaMKII was set to be maximal in the failing crest (validated by experimental data in Figure 5A), causing phosphorylation of all LTCCs there. CaMKII block eliminated L-type Ca^2+^ current appearance and EADs. These simulation results were supported by optical [Ca^2+^] recordings (Figure 4; Ca^2+^ transient oscillations in failing human cells were simulated at a pacing rate of 0.25 Hz, which could be considered similar to 0.5 Hz pacing in the rat^15^).

Although EADs in isolated cells are potential ectopic triggers, their formation does not guarantee the occurrence of arrhythmogenic triggers or arrhythmias in the whole heart. We, therefore, conducted organ-level simulations to test whether EADs resulting from HF-induced LTCC relocalization could form propagating triggers and result in arrhythmia in the heart using more physiological human heart rates, such as 1 Hz.

### Computational Model Predicts the Development of Arrhythmias in the Failing Human Ventricles

Simulation results in Online Figure XIV demonstrated that epicardial cells did not develop EADs for any of the simulated conditions, indicating that arrhythmia triggers in the failing heart were expected to develop in the endocardial layer.

**Figure 7.**
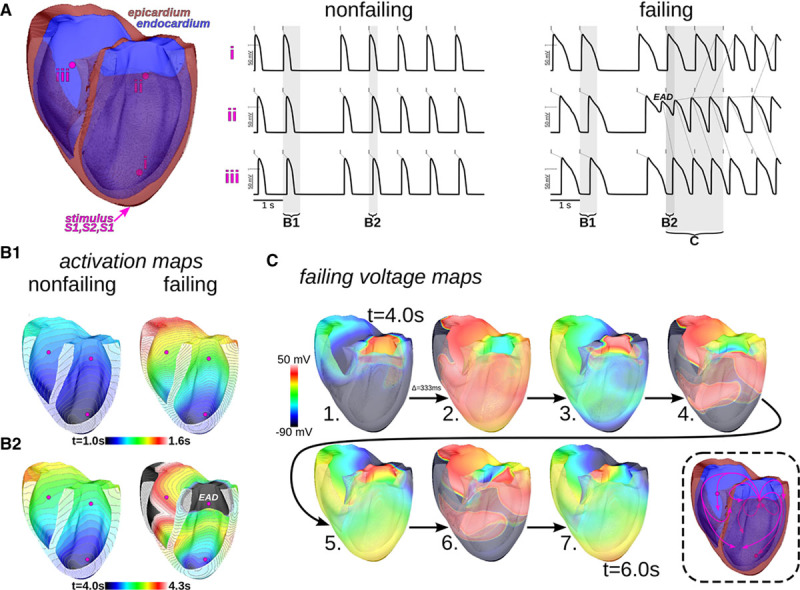
**Whole-heart simulations predict the formation of arrhythmogenic triggers and reentrant arrhythmia in human heart failure**. 1 Hz pacing with a single-skipped beat was from the apex. **A**, Action potential traces at endocardial sites “*i*–*iii*” for control and failing hearts. Small dashed gray lines relate stimuli (solid gray tick marks) to result action potentials at the sites. In the failing heart, early afterdepolarization (EAD) triggers (red, arrows) appeared near site *ii* and propagated outwardly to other sites (gray lines emanating from EADs and triggered action potentials). Vertical semitransparent bands denote time windows over which activation and voltage maps are shown in remaining panels. **B1** and **B2**, Activation maps, showing the time at which membrane voltage first crossed activation threshold, in the control and failing ventricles during the respective time windows B1 and B2 shown in **A**. **C**, Progression of activation recovery during the time window **C** shown in **A** (from 4.0 to 6.0 s, time points separated by 1/3 s). An island of EAD generating tissue can be seen near site *iii* in snapshot 1. Reentry developed after the propagation of triggered activity emanating from the EAD site (path summarized in the cartoon at bottom right; arrows show general direction of propagation and should not be taken as belonging to specific wavefronts).

The formation of arrhythmogenic triggers and reentrant arrhythmia in the failing human heart is shown in Figure 7 (1 Hz pacing with a single-skipped beat; arrhythmia did not take place without a skipped beat at this pacing rate) and in the Online Movie I. AP traces at 3 different endocardial locations are shown in Figure 7A; site “*i*” was the closest to the pacing location at the apex. In the control ventricles (Online Movie II), wavefronts propagated in an organized fashion in response to each pacing stimulus (short horizontal gray lines relate stimuli, one-to-one, to resulting APs at sites throughout the ventricles). In the failing ventricles, EAD triggers appeared near site “*ii*” and propagated (dashed gray lines from EAD triggers toward triggered APs). Activation in the control ventricles was completed within 250 ms, undisturbed by the skipped beat (Figure 7B1 and 7B2). In contrast, activation took twice as long to excite the failing ventricles. After the skipped beat pause, cells near site “*ii*” failed to repolarize, and an endocardial EAD trigger formed (Figure 7B1 and 7B2). As shown in Figure 7C, triggered activity propagated from that location, resulting in reentrant arrhythmia. The skipped beat pause had no discernable effect on subsequent activity in the control model (results not shown). Simulations also showed that triggered activity and arrhythmias did not develop in the failing ventricles when CaMKII was blocked or when crest LTCCs were made to sense dyadic [Ca^2+^], where Ca-dependent inactivation was enhanced. The whole-heart simulations, thus, demonstrated a causal link between HF-induced microdomain localization of LTCCs and arrhythmias in the failing human heart.

## Discussion

This study adds a new dimension to the understanding of cardiovascular disease, highlighting microdomain-specific changes in LTCC function, which acts in concert with well-established changes in protein expression. The major discovery of this study is that a disruption in the delicately balanced dynamic interactions between LTCCs and their cellular microenvironment can lead to pathological changes in cellular physiology and to a downstream dysfunction at the organ level. This novel concept may help to explain the molecular mechanisms of HF and other human diseases.

### Relocalization of LTCCs in HF

Here, for the first time, we provide direct evidence of the presence, in HF, of abnormally functioning LTCCs in the extradyadic space (crests) of ventricular cardiac myocytes, concurrent with changes in microdomain structure. These extradyadic LTCCs may lose the communication with the RyRs, as previous work has shown that RyR regularity and distribution do not change during HF.^38^ Only LTCCs localized in the crest had abnormally high P_o_, which contributed to the pathophysiology of HF suggesting that nanoscale changes in the location of proteins can be detrimental to their function. In fact, it has been proposed that the long open states of the LTCCs are particularly proarrhythmic in the setting of AP prolongation,^39^ suggesting that the channels that we found in the crest of failing cells can be a source of arrhythmias.

Several studies in animal models of HF^40,41^ have demonstrated a reduction in whole-cell Ca^2+^ current and in the average LTCC density, which seems to be a consequence of the profound loss of TTs, but other studies did not find changes in whole-cell Ca^2+^.^42^ Interestingly, Bryant et al^38^ showed a decreased I_Ca_ density in the TT and increased *I*_Ca_ density on the cell surface in rat HF ventricular myocytes, which supports our findings at the single-channel level. In human failing cardiac myocytes, no significant changes in LTCC density have been observed^34,36,37^; however, an impaired Cav1.2 trafficking to the TT cell membrane has been suggested.^43^ Taking into account the increased P_o_ of LTCC observed in our study (Figure 3) and in previous reports,^25^ one would expect a reduction of the number of functional channels,^5^ although we cannot exclude that a more complex mechanism could be involved. Impaired communication between LTCCs and RyRs, together with an increase in LTCC P_o_, may slow down the inactivation time, as demonstrated experimentally.^3^

It remains an open question what is the precise location of functional LTCC on the crests of sarcolemma. Are they sparsely localized on the cell surface or do they organize in special membrane microdomains forming complexes with others proteins? It is plausible that these channels are located in caveolae domains on the plasma membrane of the crest, as we recently demonstrated for extratubular LTCCs in atrial myocytes.^44^ Through the function of caveolae-based LTCCs is unknown, Makarewich et al^45^ recently demonstrated that Ca^2+^ influx through LTCCs within caveolae signaling domains can activate pathological cardiac hypertrophic signaling, and this Ca^2+^ influx can be selectively blocked without reducing cardiac contractility. Whether these channels are associated with the hyperphosphorylated LTCCs observed in our study and the extent to which they may contribute to EADs remain an open question.

### Microdomain-Dependent Changes in CaMKII Signaling

For the first time, our study reports microdomain-dependent changes in CaMKII-mediated Ca^2+^ signaling in HF (Figures 3 and 4). CaMKII, a well-known modulator of LTCCs, is upregulated under pathological conditions,^26,46^ resulting in increased LTCC P_o_.^42^ We extended these finding to show that CaMKII-dependent phosphorylation of LTCCs is increased specifically in crest microdomains (Figures 3 and 4) without affecting TT domains. CaMKII also contributed to the occurrence of abnormal calcium oscillations and lethal arrhythmias (Figures 4, 6, and 7). CaMKII is a therapeutic target, and CaMKII inhibition provides cardioprotection.^26,47^ Using experiments and simulations, we elucidated an additional mechanism for the success of CaMKII inhibition in ameliorating HF, namely the inhibition of phosphorylation of dislocated LTCCs.^48^ It has been shown that in HF, the *I*_Ca,L_ current peak density is not changed, whereas LTCCs density is decreased, suggesting an increase in the activity of the channels.^42^ We propose that the dislocated LTCCs found on the crest can be modulated by CaMKII, which could represent a new mechanism explaining this discrepancy.

CaMKII-mediated phosphorylation is an essential signaling event in triggering Ca^2+^/CaM-dependent LTCC facilitation, which requires the presence of LTCC β-subunits that can also be directly phosphorylated by CaMKII.^49^ β-subunits are upregulated in human failing myocardium and their overexpression correlates with an increase in the P_o_ of LTCCs.^50^ Thus, we suggest that in failing myocytes, where the loss of TT structure is associated with dislocation of LTCCs to the crests, increase in CaMKII activity would phosphorylate and trigger facilitation of the dislocated LTCCs via 2 factors: the weak CDI in this microdomain and direct phosphorylation of LTCC β-subunits, leading to an abnormal LTCC activity.

### Linking Subcellular Changes to Arrhythmia Propensity in HF

Using a new modeling approach spanning from stochastic LTCC gating to arrhythmogenesis at the organ level, we were able to understand how the subcellular changes are able to influence the development of arrhythmias. We found that the increase in LTCC P_o_, although CaMKII hyperactivity is specific to the crest microdomain (Figure 2), and this produces an increase of the slow inactivation of the *I*_Ca,L_ in failing cells (Figure 5B), as described previously in a stochastic model.^51^ Recently, Morotti et al^52^ using a mathematical model of rabbit AP linked a decreased CDI to an increase of the slow inactivation of the *I*_Ca,L_ and the occurrence of EADs, which was also observed in this study.

We demonstrated that in the failing ventricle, microdomain, cell- and tissue-level abnormalities act in synergy to produce whole-organ arrhythmia. It is important to note that HF is a systemic disorder in which it affects all of these hierarchical biological levels. For accurate representation of a human HF remodeling process, the membrane model of HF presented in this work used the descriptions provided in the study by Elshrif et al^53^ to define the rest of HF ion channel remodeling, outside of *I*_Ca,L_. Our study confirms that CaMKII is an important node in this network of changes in the link between HF and arrhythmias. It is involved in disease pathways although the phosphorylation of multiple key proteins, modulating ion channel functioning and affecting gene transcription, metabolism and cell survival.^54,55^ It has also been linked to the HF-associated upregulation of the late sodium current, an important contributor to EAD development.^56,57^ Although many ionic currents can contribute to EAD formation in the settings of HF-associated remodeling, *I*_Ca,L_ late appearance plays a central role in providing a regenerative inward current required for EADs to propagate,^58,59^ thereby causing triggered activity in multicellular tissue.^60^ It has been recently shown that reducing the amplitude of the noninactivating pedestal component of *I*_Ca,L_ (ie, late or window *I*_Ca,L_) effectively suppressed both H_2_O_2_− and hypokalemia-induced EADs.^61^ In nonfailing cardiac myocytes, in the settings of potassium current blockade, *I*_Ca,L_ was shown to be the main contributor to EADs formation.^62^ Conversely in HF, enhanced late sodium current may also have an important role.^57,63^ It, thus, may indicate their additive role in the enhancement of these net inward currents during the plateau phase of the AP, contributing to EAD development.

### Clinical Implications

Conventional calcium channel blockers are generally felt to be contraindicated in HF because of their negatively inotropic effects via inhibiting both peak and window *I*_Ca,L_.^59^ However, such adverse effects may relate to nonspecific targeting of LTCCs. The findings from this study can, therefore, facilitate the development of targeted and effective molecular therapies for preventing sudden cardiac death, without harmful side effects, and to steer the development of new and improved approaches to arrhythmia risk stratification of patients with HF. In the future, our assay could be used as a development platform for improved therapeutic approaches in combating HF based on the subcellular distribution of their targets. For example, selective block of the non-native LTCC pathway—directly or through subsequent regulatory proteins, including CaMKII—by novel reagents might provide an effective strategy for predicting and ameliorating the risk of sudden cardiac death in patients with cardiac disease.

### Limitation

Failing cardiac myocytes were isolated from human tissue provided with the support of NIHR Cardiovascular BRU at Royal Brompton and Harefield from explanted failing hearts only. Control cardiac myocytes were isolated from human tissue provided with the support of Hammersmith Hospital from patients who underwent mitral valve replacement surgery, with normal ejection fraction values. This indeed may introduce a difference between samples because of the regional heterogeneity within the ventricles; however, the differences in human cells between control and failing cases are consistent with the results on rat cells, which are isolated from the same region of the posterior lateral left ventricle free wall. Control human tissue for protein studies was obtained from the left ventricle free wall with the support of University of Sydney.

## Acknowledgments

We thank Dr Steven Houser for helpful discussion and critical comments on the article. We thank Peter O’Gara for cardiac myocyte isolation and Karina Zimmermann for Western blots. We are grateful to Prof Cristobal dos Remedios (University Sydney) and Prof Steve Marston for human heart muscle samples for Western blots; they were sourced through and with approval from the Australian Red Cross Blood Service and are covered by Human Research Ethics Approval from the University of Sydney (#2012/2814). Human heart failure tissue provided with the support of NIHR Cardiovascular BRU at Royal Brompton and Harefield.

## Sources of Funding

This work was supported by Wellcome Trust (J. Gorelik-WT090594; M.B. Sikkel-WT092852), British Heart Foundation (J. Gorelik-12/18/30088, A.R. Lyon-FS/11/67/28954), MRC grant (J. Gorelik-MR/L006855/1), Imperial College London Rector Award (J. Gorelik), National Institutes of Health (NIH) grants (N.A. Trayanova-R01-HL103428, R01-HL105216 and N.A. Trayanova and J. Gorelik-RO1-HL126802), NIH Director’s Pioneer Award DP1 HL123271 (N.A. Trayanova).

## Disclosures

None.

## Supplementary Material

## Novelty and Significance

### What Is Known?

- Heart failure (HF) alters cardiac function and the functionality of ion channels.
- Cells from HF models show a progressive loss of membrane structures and organization.
- L-Type calcium channels (LTCCs) are considered an important component of alterations seen in HF cells.
