# Supplementary material for: Microdomain-Specific Modulation of L-Type Calcium Channels Leads to Triggered Ventricular Arrhythmia in Heart Failure
Source: Circ Res. 2016 Sep 29;119(8):944–55. doi: 10.1161/CIRCRESAHA.116.308698 (PMC5045818; doi:10.1161/CIRCRESAHA.116.308698)
Supplement: Supplementary file 1 [file res-119-944-s001.pdf]

SUPPLEMENTAL MATERIAL

Microdomain-Specific Modulation of L-type Calcium Channels Leads to Triggered Ventricular Arrhythmia in Heart Failure

Jose L. Sanchez-Alonso, Anamika Bhargava, Thomas O'Hara, Alexey V. Glukhov, Sophie Schobesberger, Navneet Bhogal, Markus B. Sikkel, Catherine Mansfield, Yuri E. Korchev, Prakash P. Punjabi, Viacheslav .O. Nikolaev, Natalia A. Trayanova, Julia Gorelik

1. Heart failure rat model and rat cardiomyocyte isolation

Adult male Sprague-Dawley rats (250-300g) underwent proximal coronary ligation to induce chronic myocardial infarction as described before.[1, 2] Briefly, rats were anesthetized with 2% isoflurane, intubated, and ventilated after preoperative buprenorphine (0.03 mg/kg SC) injection. The thorax was shaved and sterilized with 2% w/v chlorhexidine gluconate in 70% v/v isopropyl alcohol. A left thoracotomy was performed, and the left anterior descending coronary artery was ligated with 6-0 silk. Sham ligation was used as control. Sixteen weeks later, in vivo PV analysis was performed using the 2-F Millar microconductance catheter (SPR838; Millar Instruments) via an apical approach under isoflurane (1.5%) anesthesia. Steady-state data (left ventricle end systolic and diastolic dimensions, and left ventricle ejection fraction) were recorded after 15 minutes' stabilization. Data were recorded using CHART 5.5 software (AD Instruments) and analyzed off line using PVAN 3.6 software (Millar Instruments). Hearts were explanted, weighed, and prepared for cell isolation.

This heart failure model recapitulates many features of chronic heart failure in patients including adverse remodeling of the organ, characterized by left ventricle and left atria dilatation, reduced ejection fraction, raised filling pressures and elevated serum natriuretic peptides.[2-4] In particular, heart failure rats exhibited left ventricle dilatation, reduced ejection fraction and increase in the cell length (**Online Figure I**). Ventricular cells from this model have been studied extensively and are well characterized at structural, biochemical, molecular, Ca^{2+} handling, and electrophysiological levels.[2, 4, 5]

Ventricular cardiomyocyte isolation was done as previously described.[1] Briefly, Sprague-Dawley rats (150–250 g) were anesthetized with 5% isoflurane-95% O_2 and then killed by cervical dislocation. Hearts were fast extracted and placed in Tyrode solution containing in (mmol/L): 140 NaCl, 6 KCl, 1 $MgCl_2$, 1 $CaCl_2$, 10 glucose and 10 HEPES, adjusted to pH 7.4 with 2 mmol/L NaOH. Using aortic cannulation with the Langendorff setting, the hearts were perfused with Tyrode solution for 5 min, then with low Ca^{2+} solution containing in (mmol/L): 120 NaCl, 5.4 KCl, 5 $MgSO_4$, 5 sodium pyruvate, 20 glucose, 20 taurine, 10 HEPES, 5 nitrilotriacetic acid, and 0.04 $CaCl_2$, adjusted to pH 6.96 with 2 mmol/L NaOH for 5 min, and finally for 10 min with enzyme solution containing in (mmol/L): 120 NaCl, 5.4 KCl, 5 $MgSO_4$, 5 sodium pyruvate, 20 glucose, 20 taurine, 10 HEPES, and 0.2 $CaCl_2$, pH 7.4 with collagenase (1 mg/ml; Worthington) and hyaluronidase (0.6 mg/ml; Sigma-Aldrich).

Cardiomyocytes were plated on dishes coated with laminin and left to stick to bottom for at least 45 minutes before experiments. Cardiomyocytes were used on the same day as isolation.

2. Patients groups and human cardiomyocyte isolation

To control for factors of regional-dependent heterogeneity, the presence of acute ischemia, and ischemic injury, the current study was conducted on dilated end-stage cardiomyopathic human hearts (n=6, patients, average age 48±5 years, two females and fourth males, **Online Table I**) with the approval from Bromton Harefield & NHLI Research Ethic Committee (Ref 01-194). For comparison, we used ventricular left-over specimens obtained with the consent from non-failing patients (n=5, average age 69±2 years, two females and three males, **Online Table II**) during valve replacement procedure at Hammersmith Hospital, Imperial College London, London, UK. Although non-failing control patients were complicated by various factors, including age, early stage hypertrophy, atrial fibrillation, and coronary disease, they possessed normal left ventricle function (ejection fraction >60%).

Failing ventricular cardiomyocytes were isolated from the apical section of the posterior-lateral left ventricle free wall by enzymatic digestion as previously described.[2] Briefly, individual specimens were transferred to ice-cold calcium free Krebs-Ringer saline solution consisting of (in g/L): 7.012 NaCl, 0.402 KCl, 1.332 $MgSO_4$, 0.55 Pyruvate, 3.603 Glucose, 2.502 Taurine, 2.383 HEPES, 1.286 Nitrillotriacetic Acid; pH = 6.96. Connective and adipose tissue were removed and approximately 500 mg of myocardial tissue was minced

with razor blades in small cubes (approx. 1-2 mm^3). Then, the tissue pieces were washed with fresh Ca^{2+}-free Krebs-Ringer solution 3 times for 3 min each at 37°C. After wash, cardiac tissue was incubated for 25 min in 10 ml of Krebs-Ringer solution containing (in g/L): NaCl 7.012, KCl 0.402, $MgSO_4$ 1.332, Pyruvate 0.55, Glucose 3.603, Taurine 2.502, HEPES 2.383; pH = 7.4, supplemented with 200 nM $CaCl_2$ and Proteinase type XXIV (0.36mg/ml; Sigma-Aldrich) under gentle agitation. The partially digested tissue was transferred to 10 ml of Krebs-Ringer saline supplemented with collagenase type XIV (1mg/ml Sigma-Aldrich). The tissue was incubated thrice with this solution for 10 min each at 37°C with gentle agitation. Usually, cardiomyocytes were visible by phase contrast light microscopy after the first incubation step, with the biggest amount of cells after the second incubation step. After each incubation step, the supernatants were transferred to a tube and centrifuged at 600 rpm for 3 min. The pellets were re-suspended in 2-3 mL of Krebs-Ringer solution. After isolation, human cardiomyocytes were plated following the same protocol as rat cardiomyocytes. Non-failing control human ventricular cardiomyocytes were isolated from the left ventricle papillary muscles.

3. T-tubule labelling and analysis

Di-8-ANEPPS was excited at a wavelength of 488 nm and confocal images were taken at 63x magnification using a Zeiss LSM-780 inverted confocal microscope. The 40x5 microns size area inside the sarcolemma that did not coincide with the cell nuclei was chosen, automatically thresholded into binarised, and the TT density was determined as ratio of black pixels versus white pixels. The TT regularity was calculated through a single dimension Fourier transformation using a custom-written macro for Matlab (The MathWorks, Inc., Natick, MA, USA) as described before.[6]

For the visualization of the TT network structure in combination with SICM, cardiomyocytes were stained with the fluorescent dye Di-8-ANEPPS (10µM) for 1 min. Di-8-ANEPPS was excited at a wavelength of 473 nm with a StradusTM473 laser (Vortran) and confocal images were taken at 100x magnification using a Photomultiplier Detection System (PTI).

4. Super-resolution scanning patch-clamp with pipette clipping modification

This technique combines scanning ion conductance microscopy (SICM) and patch-clamp electrophysiology with a pipette clipping modification to increase the throughput of recording

ion channel activity from cell surface microdomains. SICM uses a sharp pipette (~100 nm inner diameter, ID, 100 MΩ resistance) as a scanning probe to generate high resolution topography images of live cells.[7] SICM is based on the principle that the ion current through the electrolyte-filled micropipette is partially occluded when the pipette approaches the surface of a cell.[8] Therefore, the position of the tip of the pipette relative to the cell surface strongly influences the ion current through the pipette. This ion current is digitized and fed into the feedback and scan control system which provides the feedback signal to control the vertical position of the pipette keeping the pipette-sample separation constant. The pipette raster scans the sample and generates a 3D topography image of the cell surface.

Next, to clip the pipette tip to generate a wider pipette necessary to increase the chances of getting an ion channel, the pipette is positioned above a free surface away from cells. Then, moving down with high velocity the pipette is allowed to impact onto the surface and the tip of the pipette clips in a controlled manner.[7] This is possible because at a high enough velocity of approaching the surface the feedback control of the scanning system cannot generate enough upward movement of the piezo-drive quickly enough to avoid contact with the surface. With this method at 500 nm/ms the pipette tip is clipped to approximately ~350 nm ID, 30 MΩ resistance. The pipette is then positioned back onto the cellular microdomain of choice (in this case T-tubule or crest) taking help of the coordinates from the image acquired before clipping. The feedback is turned off; pipette is lowered until it touches the cell surface and a very gentle suction is applied to form a gigaseal (**Online Figure II**). Cell-attached single channel recordings are then performed by the conventional patch-clamp technique.

5. Electrophysiological recordings

5.a. Cell attached LTCC

Cell-attached patch-clamp recordings of single LTCC currents were performed at room temperature using the following solutions; external solution containing in (mmol/L): 120 K-gluconate, 25 KCl, 2 MgCl2, 1 $CaCl_2$, 2 EGTA, 10 Glucose, 10 HEPES, pH 7.4 with NaOH, ~290 mOsm; internal recording solution containing in (mmol/L): 90 $BaCl_2$, 10 HEPES, 10 Sucrose, pH 7.4 with TEA-OH, ~250 mOsm. The pipette used for cell attached recordings had a typical resistance of approximately 30 MΩ. Currents were recorded using Axopatch 200A amplifier (Axon Instruments, Foster City, CA, USA), controlled and monitored using pClamp software version 10 (Axon Instruments). Single LTCCs were identified and

characterized by their voltage dependent properties. To generate a current-voltage (I–V) relationship, the membrane under the patch was held at a voltage of -80 mV and voltage pulses were applied from -30 to +30 mV in the incremental steps of 10 mV. Analysis was performed as previously described.[7] Single channels were sampled at 10 kHz and filtered at 2 kHz (- 3 dB, 8-pole Bessel). Single channel data were analysed using Clampfit version 10.2. A liquid junction potential, calculated to be -16.7 mV, was corrected from the data shown.

Every time a channel is recorded a minimum of 100 sweeps at -6.7 mV (after liquid junction potential correction) and 3 full protocols at different voltage steps were done. All the sweeps are checked for the presence of more than one channel. When no second level of spikes is observed in any of the sweeps the recording is classified as one channel. Overall, from 89 recordings showing activity in this work, 70 (79%) of them were classified as one channel, 13 (14%) show two channels, 5 (6%) triple channels, and only in one occasion (1%) the recording showed 4 channels, and this is similar between TT and Crest regions. From 22 recordings of human cells 64% were classified as one channel versus rat cells where from 67 recordings 84% were classified as one channel. Seal data is showed in detail on **Online Table III**.

Occurrence of LTCCs was calculated as the percentage of recording showing activity versus the total number of recordings, higher occurrence can be interpreted as a higher density of channels on that specific location or group.

The open probability (Po) was averaged from 10-20 sweeps at -6.7 mV for each cell. Each cell was recorded only once, and only one value of Po per cell was used. The total number of channels in the recording was input into pClamp software to calculate the Po and the peak current (calculated as the overall average current) of one single channel.

5.b. Action potential recordings

APs were recorded from isolated LV rat cardiomyocytes using the current-clamp configuration of the patch-clamp technique (Figure S12) with the external recording solution containing (mmol/L): 120 NaCl, 5.4 KCl, 0.2 $CaCl_2$, 5 $MgSO_4$, 10 HEPES, 20 glucose, 5 pyruvate sodium, 20 Taurine, pH 7.4 with NaOH, ~300 mOsm, and the internal pipette solution containing (mmol/L): 120 KCl, 5 EGTA, 10 HEPES, 0.5 MgCl2, 1 $CaCl_2$, 5 Mg-ATP, pH 7.3 with KOH, ~290 mOsm. Currents were recorded using Multiclamp 700B amplifier (Axon Instruments, Foster City, CA, USA), controlled and monitored using pClamp software version 10 (Axon Instruments). Patch pipettes had typical resistances of 8-10 MΩ.

The bath was connected to the ground via an Ag–AgCl pellet. Data were sampled at 10 kHz. All recordings were performed at 33-35°C. APs were elicited with 5-ms current pulses at 1Hz. AP characteristics were analysed using pClamp software.

5.c. Whole-cell ICaL

Macroscopic Ca2+ currents were recorded using the whole-cell patch-clamp technique (Figure S13) with the external recording solution containing (mmol/L): 1 $CaCl_2$, 0.5 $MgCl_2$, 5 HEPES, 140 choline chloride, 5 CsCl, 5.5 glucose, pH 7.4 with CsOH, ~305 mOsm, and the internal pipette solution containing (mmol/L): 130 Cs-methanesulphonate, 11 EGTA, 10 HEPES, 2 $MgCl_2$, 5 Mg-ATP, 0.3 Na-GTP, pH 7.2 with CsOH, ~290 mOsm. Currents were recorded using Multiclamp 700B amplifier (Axon Instruments, Foster City, CA, USA), controlled and monitored using pClamp software version 10 (Axon Instruments). Patch pipettes had typical resistances of 1–2 MΩ. The bath was connected to the ground via an Ag–AgCl pellet. Data were low-pass filtered at 2 kHz using the built-in Bessel filter of the amplifier and sampled at 10 kHz. The amplifier was also used for capacitive transient and series resistance compensation between 60 and 70% on each cell. Leak subtraction of leakage currents was performed with Clampfit during off-line analysis. All recordings were performed at 33-35°C. I–V relationships were obtained by holding the cells at a potential of −50 mV before applying 100 ms pulses to potentials from −70 to +40 mV every 5s in 10 mV increments. I–V relationships were fitted with the modified Boltzmann equation, $I = [G_{max} \times (Vm - Erev)]/\{1 + \exp[(Vm - V0.5a)/Ka]$, where Vm is the test potential, V0.5a is the half-activation potential, Erev is the extrapolated reversal potential, G_{max} is the maximum slope conductance and Ka reflects the slope of the activation curve.

6. Single Channel Human L-type Ca^{2+} Current ($I_{Ca,L}$)

6.a. Markov Formulation

Human ventricular cell electrophysiological behavior was represented by the O'Hara-Rudy model,[9] selected because L-type Ca^{2+} current, $I_{Ca,L}$, in the model was based on un-diseased human data, and because accurate representations of heart failure ion channel remodeling in transmural cell types of the model are available. The O'Hara-Rudy $I_{Ca,L}$

follows the Hodgkin-Huxley (HH) formalism. However, we needed to model stochastic single channel behavior so that we can determine the channel open probability (P_o) for comparison with human experimental data. We thus converted the HH $I_{Ca,L}$ model to its Markov-equivalent representation.

Online Figure IV shows the Markov equivalent of the O'Hara-Rudy $I_{Ca,L}$. There are 32 states in each of the CaMKII un-phosphorylated and CaMKII phosphorylated modes (64 total states). In each mode, there are 6 open states. Nested cubes in each mode are identical, except for open states, and include activation/deactivation by the “d” gate (right/left) and inactivation/recovery by the “f” (up/down) and “fca” (in/out of page) gates. The inner- and outermost pairs of nested cubes are connected by the slow recovery “jca” gate. The gate “nca” is Ca^{2+}-sensitive and specifies whether inactivation is voltage (“f”), or Ca^{2+} (“fca”) driven. The innermost cube connects with the third cube, and the second cube connects with the outermost one via the “nca” gating. Thus, the four discrete combinations of “jca” and “nca” define the four nested cube (from innermost to outermost cube: jca=1, nca=0; jca=0, nca=0; jca=1, nca=1; jca=0, nca=1).

Both “f” and “fca” gates have fast and slow gating modes. CaMKII un-phosphorylated channels inactivate in both fast and slow gating modes. Inactivation occurs via the slow mode only for channels phosphorylated by CaMKII.

6. b. Stochastic Sweeps using Gillespie Exact Algorithm

To evolve the channel gating in response to a 1-s voltage change step to -6.7 mV (from resting state, marked *), we used the Gillespie Exact Algorithm, implemented in Matlab (The Mathworks Inc.). The built-in pseudo-random number generator function, “rand”, was seeded to the system clock and used twice at each state change step, as required by the algorithm. Example single channel sweeps are shown in **Online Figure VIII**.

6. c. Reverting to Hodgkin-Huxley Formalism

Once single channel current simulation results were generated and validated with experimental P_o measurements, the $I_{Ca,L}$ model was reverted back to an HH formulation. The HH formulation retained the CaMKII mode definitions and the behavior of the equivalent Markov version, but could be numerically integrated rapidly and with ease in cell (the O'Hara-Rudy model) and tissue simulations. Failing Crest LTCCs were assumed to operate in CaMKII phosphorylated mode, based on experimental findings. Their inactivation was strictly via the slow gating mode. Failing LTCCs in TTs and control LTCCs were sensitive to

standard CaMKII levels in the cell, and so inactivation was both fast and slow. Ensemble current computed by summation of single channel sweeps matched the deterministic HH current, as expected (**Online Figure V**).

7. Models of Human Control Nonfailing and Failing Ventricular Myocytes

7. a. Fast Na^+ Current (I_{Na})

As noted online in the PLoS Computational Biology comments associated with the O'Hara et al. 2011 publication of the O'Hara-Rudy ionic model,[9] we replaced the original I_{Na} with the formulation used in the ten-Tusscher et al human ventricular cell model.[10] The ten-Tusscher et al formulation was based on the same set of human experimental data. The ten-Tusscher et al myocyte model and its I_{Na} have been used extensively to simulate action potential propagation in tissue (e.g. [11, 12]).

7. b. Sub-sarcolemmal Volume

In the Grandi et al human cell model,[13] SL volume was 0.0308 times that of the bulk myoplasm and 3.7106 times that of the dyad (or junctional) volume. However, bulk to dyad volume ratio is different in the Grandi et al and O'Hara-Rudy models. The Grandi et al model definition could be directly adopted by the O'Hara-Rudy model as either the ratio of bulk myoplasm volume to SL volume, or as dyadic volume to SL volume. In this study, SL volume in the O'Hara-Rudy model was set to be the average of these two options (0.04755 times the whole cell volume).

As was done by Grandi et al and Shannon et al, we set the flux rate between SL and myoplasm to be 0.2213 times that between dyad and SL. We assumed that the geometry of the dyad/SL interface was identical to the original O'Hara-Rudy dyad/myoplasm interface.

Ca^{2+}-dependent inactivation (CDI) of $I_{Ca,L}$ occurs regardless of Ca^{2+} origin, whether from Ca^{2+} release from RyRs in the dyadic volume or from the channel's own flux. The original O'Hara-Rudy myocyte model did not include a sub-sarcolemmal (SL) volume and thus did not allow for Ca^{2+} accumulation near the intracellular mouth of Crest LTCCs. In the human ventricular cell model by Grandi et al.,[13] an SL volume was included (originally incorporated in the Grandi et al model predecessor, a rabbit ionic model by Shannon et al [14]). We incorporated SL volume and related fluxes in the O'Hara-Rudy myocyte model based on the work of Grandi et al and Shannon et al.[13, 14]

As was done by Grandi et al and Shannon et al, we set the flux rate between SL and myoplasm to be 0.2213 times that between dyad and SL. We assumed that the geometry of the dyad/SL interface was identical to the original O'Hara-Rudy dyad/myoplasm interface.

7. c. LTCC Current Density at TT and Crest Locations

The original O'Hara-Rudy model included LTCCs exclusively at TT sites with dyadic intracellular face. In the present simulations, LTCCs in the TTs sensed and contributed to dyadic Ca^{2+}; channels newly added to the Crest sensed and contributed to SL Ca^{2+}. Channel kinetics and all equations governing behavior were identical in TT and crest. That is, changes in current function were solely based on extrinsic environmental changes, not intrinsic changes in kinetics.

In each of TT and crest locations, LTCC permeability, PCa, representing whole-cell current density in TTs (PCa_{TT}) and crest (PCa_{Crest}) needed to be defined. We utilized experimental data obtained in this study to assign values to PCa_{TT} and PCa_{Crest} in control and failing human cells. The data used were: % occurrence of LTCCs, LTCC single channel current amplitude, and in failing cells, the degree of TT loss. Thus, we arrived at the following values for fractional PCa (fPCa) in control cells: $fPCa_{control,TT}$ = 0.75 and $fPCa_{control,Crest}$ = 0.25 (see manuscript **Figure 2A**). The original O'Hara-Rudy model PCa value was multiplied by these fractions to get accurate current density at TTs and crest in control myocytes.

Values for fractional PCa (fPCa) in control cells were: $fPCa_{control,TT}$ = 0.75 and $fPCa_{control,Crest}$ = 0.25 (see manuscript **Figure 2A**). The original O'Hara-Rudy model PCa value was multiplied by these fractions to get accurate current density at TTs and crest in control myocytes.

In failing human cells, our experimental findings (data not shown) indicated that LTCC single channel amplitude was slightly, but statistically significantly greater in TTs (1.2 ± 0.07 -fold greater) and slightly but statistically significantly lower at crest sites (0.85 ± 0.06 times) when compared to control TT amplitude, the later being our reference amplitude. Thus, the LTCC % occurrence (as a fraction of total LTCC occurrence), (manuscript Figure 2A), was multiplied by these single channel amplitude factors (1.2 and 0.85 for TT and Crest, respectively). For failing Crest, this was $fPCa_{failing,Crest}$ = 0.75*0.85 = 0.63.

To arrive at a value for $fPCa_{failing,TT}$, an additional factor accounting for the degree of TT loss was incorporated. This was necessary because TT loss reduces whole cell density of LTCC TT channels, which lowers $fPCa_{failing,TT}$. Loss of TTs in failing human myocytes was

the average of fractional TT density (failing/control = 0.51 ± 0.08) and fractional Z-groove ratio (failing/control = 0.41 ± 0.06, average of 0.54). Thus, $fPCa_{failing,TT}$ = 0.75*1.2*0.54 = 0.486.

Finally, experimental evidence from human myocytes has indicated (see for example [15]) that HF ion channel remodeling does not have an effect on peak I_{CaL}. Therefore, we adjusted the whole cell LTCC peak current in failing myocytes to match that of control.

In summary:

$PCa_{control,TT} = 0.75$

$fPCa_{control,Crest} = 0.25$

$PCa_{failing,TT} = 0.3846$

$fPCa_{failing,Crest} = 0.4985$

7. d. Na^+/Ca^{2+} Exchanger (NCX)

The original O'Hara-Rudy model assumes that 20% of NCX channels reside on the surface membrane. In the present simulations, the percent of NCX residing at crest sites (i.e. non-TT-residing NCX) was increased in accordance with the degree of TT loss, assuming that NCX migrates to the Crest in a way similar to LTCCs migration following TT loss. Since the value for the fraction of intact TTs was 0.54 (see above), 46% (100%-54%) of the original 80% of NCX residing in TTs were relocated to the Crest in failing myocytes. The importance of accounting for NCX re-distribution following TT degradation arises from the fact that it affects concentrations in the SL volume.

7. e. "Orphaned" RyRs

With TT loss, the association between LTCCs and RyRs is also lost, affecting Ca^{2+} induced Ca^{2+} release. The "orphaned" RyRs generate poorly coordinated Ca^{2+} release events.[16] This was represented in the HF myocyte model by defining the I_{CaL} trigger as $I_{CaL,TT}$ and by restricting RyR flux ("Jrel") to the portion of remaining TTs (i.e. to 54% of the original number of TTs). This resulted in weakening of the trigger/response coupling between LTCCs and RyRs in failing cells, and in turn, affected LTCCs either directly or indirectly based on Ca^{2+} in their TT of Crest location, respectively.

A more biophysically accurate and complete representation of RyR orphaning requires high sub-cellular spatial resolution not captured by the common pool dyadic volume in the O'Hara-Rudy model. Such representations are required for simulation of Ca^{2+} waves,

which can occur in failing myocytes, but are not the focus of this study. The above, more phenomenological representation of RyR orphaning kept the focus of this research on LTCCs in the failing cell, and enabled implementation of the myocyte model in an organ-level electrophysiological model to study the downstream electrophysiological consequences. i.e. arrhythmogenesis.

7. f. Heart Failure Remodeling

A recent publication provided a comprehensive literature review of heart failure remodeling data in the human myocyte and a summary of how the data have been used in previous computational models.[15] The publication used the O'Hara-Rudy model and presented endocardial and epicardial transmural definitions of heart failure ion channel remodeling, implemented as scaling of conductances to best represent the human dataset. We used the descriptions provided in Elshrif et al. ([15], **table 2**) to define the rest of HF ion channel remodeling (outside of $I_{Ca,L}$) in our model.

Holzem et al.[17] examined rapid delayed rectifier current (I_{Kr}) remodeling, providing new human data and a human myocyte model based on the Grandi et al framework; simulations of myocyte behavior in HF best matched experimental data when I_{Kr} magnitude was reduced by 50%. We used this value for I_{Kr} magnitude remodeling in the failing myocyte model in the present study.

A ~3-fold increase in CaMKII activity has been reported in human failing versus control cells [18]. In the failing myocyte model we thus assumed that CaMKII at Crest/SL sites was maximal, while in the rest of the failing cell it was multiplied by a factor of 3.

References

1. Vescovo G, Jones SM, Harding SE, Poole-Wilson PA. Isoproterenol sensitivity of isolated cardiac myocytes from rats with monocrotaline-induced right-sided hypertrophy and heart failure. *J Mol Cell Cardiol*. 1989;21:1047-1061.

2. Lyon AR, MacLeod KT, Zhang Y, Garcia E, Kanda GK, Lab MJ, Korchev YE, Harding SE, Gorelik J. Loss of t-tubules and other changes to surface topography in ventricular myocytes from failing human and rat heart. *Proc Natl Acad Sci USA*. 2009;106:6854-6859.

3. Yanni J, Tellez JO, Maczewski M, Mackiewicz U, Beresewicz A, Billeter R, Dobrzynski H, Boyett MR. Changes in ion channel gene expression underlying heart failure-induced sinoatrial node dysfunction. *Circulation Heart Fail*. 2011;4:496-508.

4. Lyon AR, Bannister ML, Collins T, Pearce E, Sepehripour AH, Dubb SS, Garcia E, O'Gara P, Liang L, Kohlbrenner E, Hajjar RJ, Peters NS, Poole-Wilson PA, Macleod KT, Harding SE. Serca2a gene transfer decreases sarcoplasmic reticulum calcium leak and reduces ventricular arrhythmias in a model of chronic heart failure. *Circulation Arrhythm Electrophysiol*. 2011;4:362-372.

5. Lyon AR, Nikolaev VO, Miragoli M, Sikkel MB, Paur H, Benard L, Hulot JS, Kohlbrenner E, Hajjar RJ, Peters NS, Korchev YE, Macleod KT, Harding SE, Gorelik J. Plasticity of surface structures and beta(2)-adrenergic receptor localization in failing ventricular cardiomyocytes during recovery from heart failure. *Circulation Heart Fail*. 2012;5:357-365.

6. Ibrahim M, Navaratnarajah M, Siedlecka U, Rao C, Dias P, Moshkov AV, Gorelik J, Yacoub MH, Terracciano CM. Mechanical unloading reverses transverse tubule remodelling and normalizes local Ca^{2+}-induced Ca^{2+} release in a rodent model of heart failure. *Eur J Heart Fail*. 2012;14:571-580.

7. Bhargava A, Lin X, Novak P, Mehta K, Korchev Y, Delmar M, Gorelik J. Super-resolution scanning patch clamp reveals clustering of functional ion channels in adult ventricular myocyte. *Circ Res*. 2013;112:1112-1120.

8. Novak P, Li C, Shevchuk AI, Stepanyan R, Caldwell M, Hughes S, Smart TG, Gorelik J, Ostanin VP, Lab MJ, Moss GW, Frolenkov GI, Klenerman D, Korchev YE. Nanoscale live-cell imaging using hopping probe ion conductance microscopy. *Nature Methods*. 2009;6:279-281.

9. O'Hara T, Virag L, Varro A, Rudy Y. Simulation of the undiseased human cardiac ventricular action potential: Model formulation and experimental validation. *PLoS Computational biology*. 2011;7:e1002061.

10. ten Tusscher KH, Noble D, Noble PJ, Panfilov AV. A model for human ventricular tissue. *Am J Physiol Heart Circ Physiol*. 2004;286:H1573-1589.

11. Ten Tusscher KH, Hren R, Panfilov AV. Organization of ventricular fibrillation in the human heart. *Circ Res*. 2007;100:e87-101.

12. Bayer JD, Narayan SM, Lalani GG, Trayanova NA. Rate-dependent action potential alternans in human heart failure implicates abnormal intracellular calcium handling. *Heart Rhythm*. 2010;7:1093-1101.

13. Grandi E, Pasqualini FS, Bers DM. A novel computational model of the human ventricular action potential and ca transient. *J Mol Cell Cardiol*. 2010;48:112-121.

14. Shannon TR, Wang F, Puglisi J, Weber C, Bers DM. A mathematical treatment of integrated ca dynamics within the ventricular myocyte. *Biophys J*. 2004;87:3351-3371.

15. Elshrif MM, Shi P, Cherry EM. Representing variability and transmural differences in a model of human heart failure. *IEEE*. 2015;19:1308-1320.

16. Song LS, Sobie EA, McCulle S, Lederer WJ, Balke CW, Cheng H. Orphaned ryanodine receptors in the failing heart. *Proc Natl Acad Sci USA*. 2006;103:4305-4310.

17. Holzem KM, Gomez JF, Glukhov AV, Madden EJ, Koppel AC, Ewald GA, Trenor B, Efimov IR. Reduced response to i blockade and altered herg1a/1b stoichiometry in human heart failure. *J Mol Cell Cardiol*. 2015.

18. Kirchhefer U, Schmitz W, Scholz H, Neumann J. Activity of cAMP-dependent protein kinase and Ca^{2+}/calmodulin-dependent protein kinase in failing and nonfailing human hearts. *Cardiovasc Res*. 1999;42:254-261.

19. Glukhov AV, Fedorov VV, Lou Q, Ravikumar VK, Kalish PW, Schuessler RB, Moazami N, Efimov IR. Transmural dispersion of repolarization in failing and nonfailing human ventricle. *Circ Res*. 2010;106:981-991.

Online Figure I.

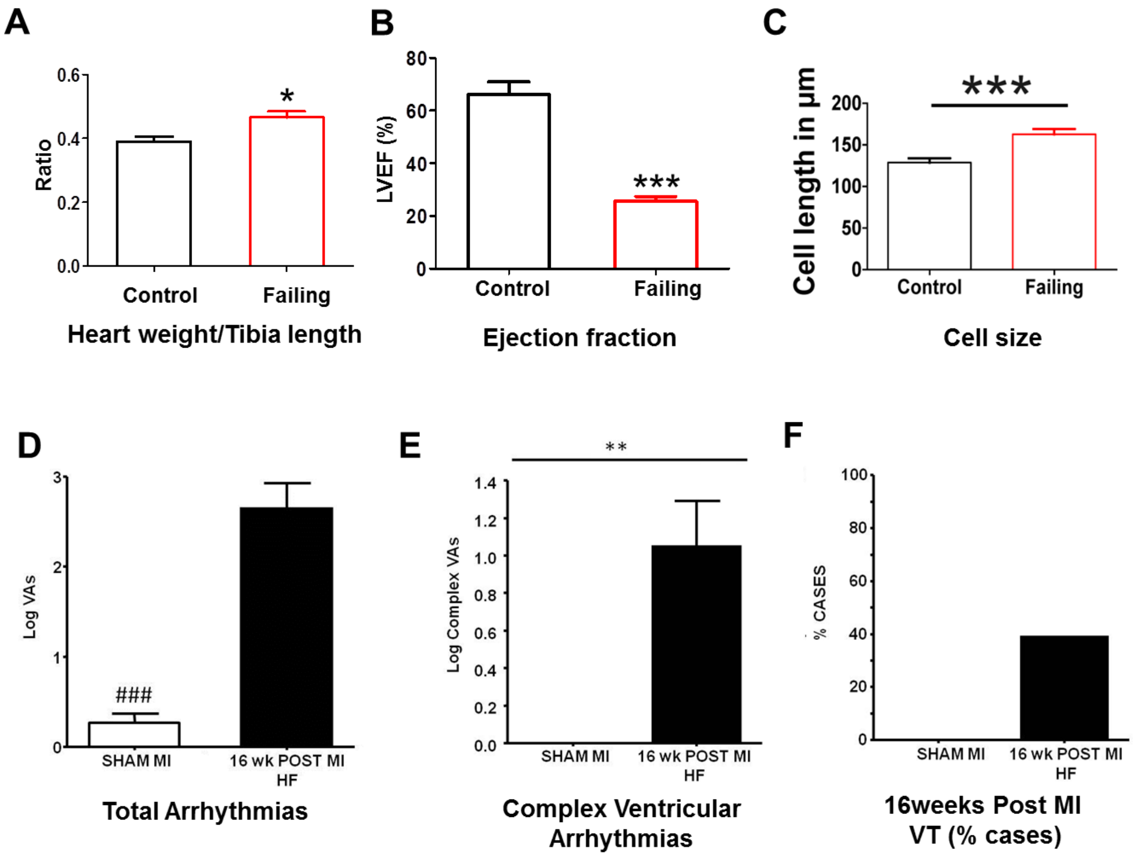

Online Figure I. Characteristics of the rat model of heart failure. (A) Heart weight to tibia length ratio was significantly increased in heart failure (control n=8 vs. failing n=16, $P<0.05$). **(B)** Ejection fraction was significantly decreased in heart failure (LVEF, left ventricular ejection fraction, control n=6 vs failing n=14, $P<0.001$). **(C)** Cell size was significantly increased in heart failure (control n=20 vs. failing n=20, $P<0.001$). **(D)** Total arrhythmias, ($P<0.001$) **(E)** Complex ventricular arrhythmias ($P<0.01$) **(F)** VT % cases.

Online Figure II.

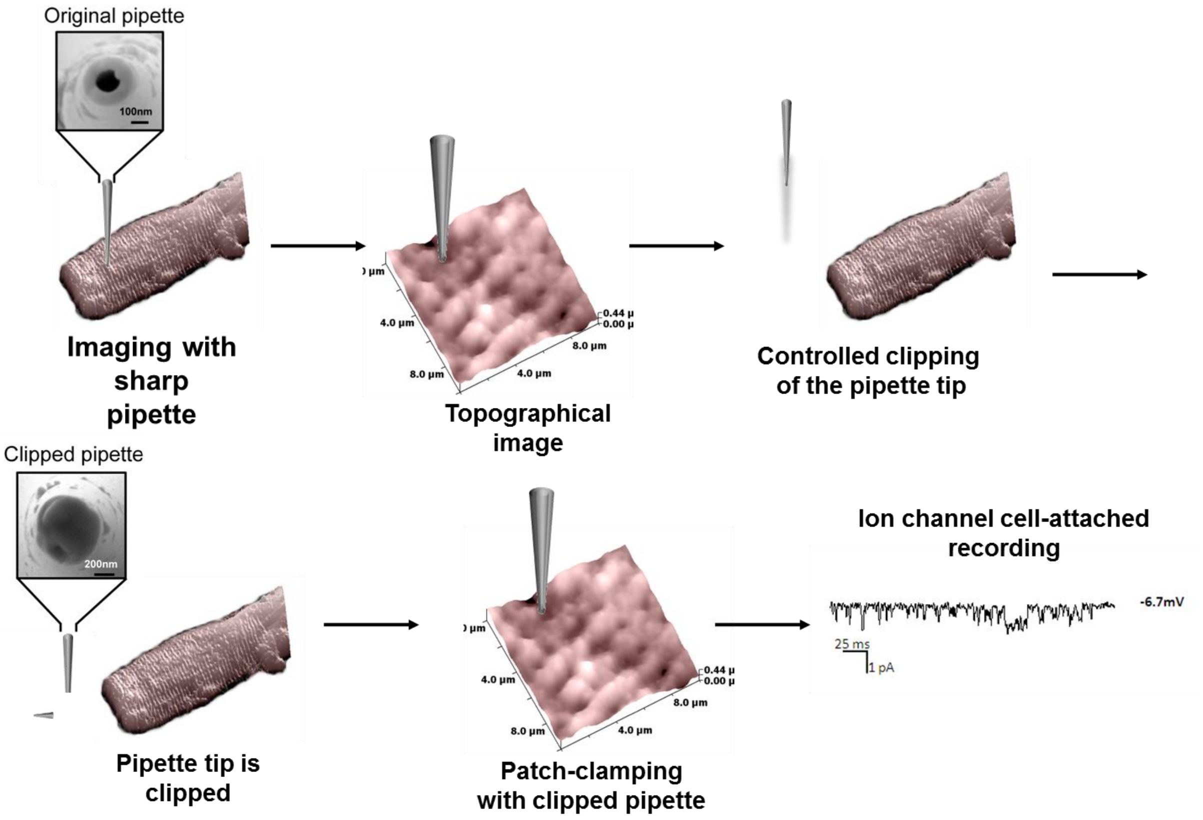

Online Figure II. Super-resolution scanning patch-clamp method. A topographical image of the cardiomyocyte generated by SICM with a 100 nm nanopipette. The inner tip diameter of the nanopipette is widened to ~350 nm by controlled clipping to increase the area of attachment. First, the pipette was navigated to an area free of cardiomyocytes, the fall rate was increased and pipette was allowed to impact on surface. As a result the pipette breaks its tip and increases its diameter because of the conical shape of the pipette. Pipette tip breaking resulted in stepwise increases of the pipette current as its resistance dropped. The breaking was automatically stopped by returning the fall rate to baseline (60 nm/ms) once the pipette current reached a desired level. After clipping, the pipette is lowered to a specific location (TT or crest) and a gigaseal is established. Single ion channels are recorded in cell-attached mode. Insets show the pipette tip size before and after clipping.

Online Figure III.

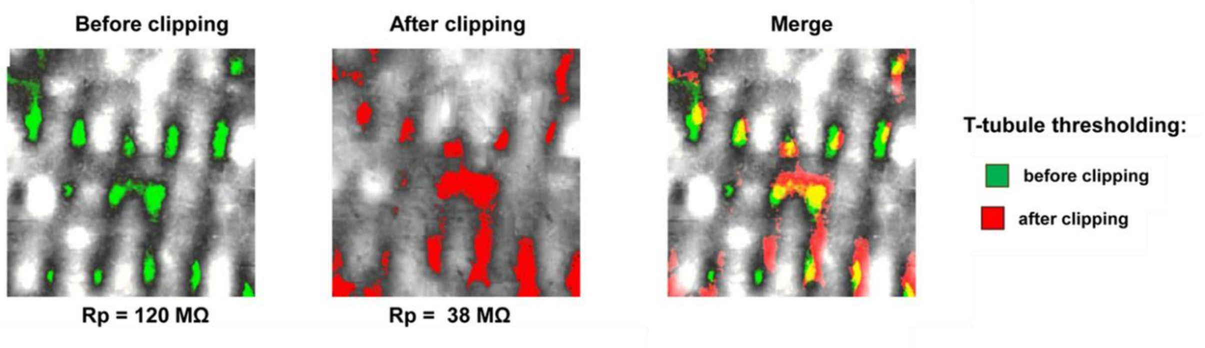

Online Figure III. Clipping procedure does not change pipette position. T-tubule location on the surface scan before (*left*) and after (*middle*) clipping. After clipping the pipette resistance (Rp) reduced from 120 MΩ to 38 MΩ. Surface deepness thresholding was used to visualize location of T-tubules before (green) and after clipping (red). Because the pipette was held vertically at all times, *x, y* coordinates of the pipette tip did not change.

Online Figure IV.

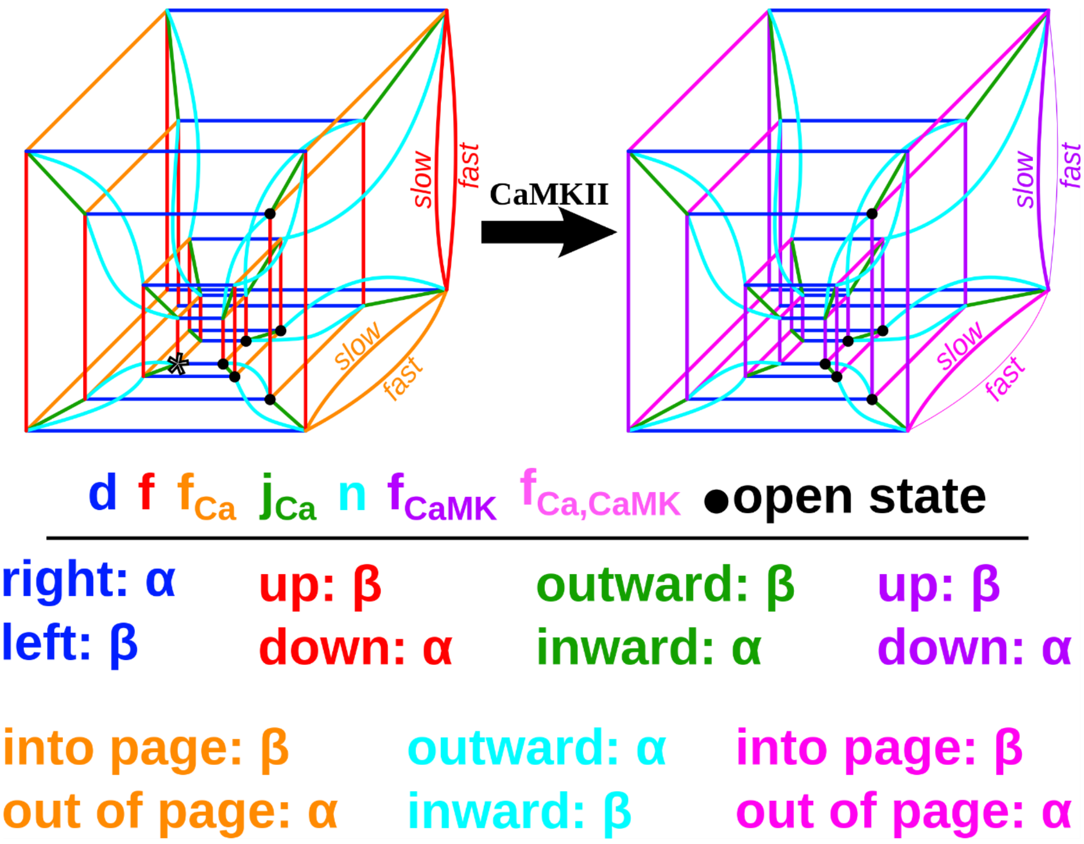

Online Figure IV. State diagram of the Markov model equivalent of the O'Hara-Rudy Hodgkin-Huxley (HH)-formalism-based L-type Ca^{2+} current formulation. Vertices of nested cubes on the left show connected states in the non-CaMKII-phosphorylated model. To the right are CaMKII-phosphorylated model states, distinguished by 2.5-fold slower inactivation ("f_{CaMK}" and "$f_{Ca,CaMK}$" in purple and pink are slower than "f" and "f_{Ca}" in red and orange, left). When CaMKII phosphorylated, inactivation followed "slow" rather than "fast" paths (indicated by thick "slow" and thin "fast" connections in purple and pink). Colors and directional descriptors below indicate correspondence between the HH-based gates and Markov state transitions, as drawn in the diagram. Open states are marked by black dots.

Online Figure V.

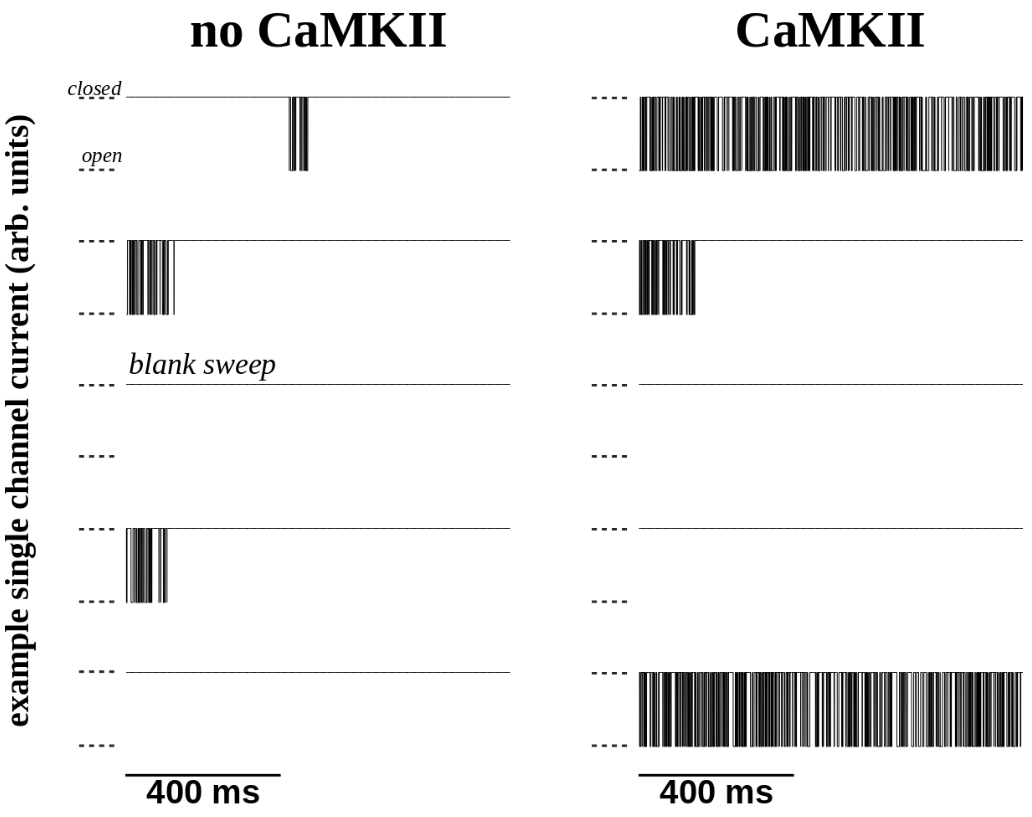

Online Figure V. Examples of random single channel current sweeps. The Gillespie Exact Monte Carlo Algorithm was used to calculate stochastic gating in response to a 1 s voltage step to -6.7 mV. Sweeps show early and infrequent openings without CaMKII (left column of sweeps) and frequent and late openings with CaMKII phosphorylation.

Online Figure VI.

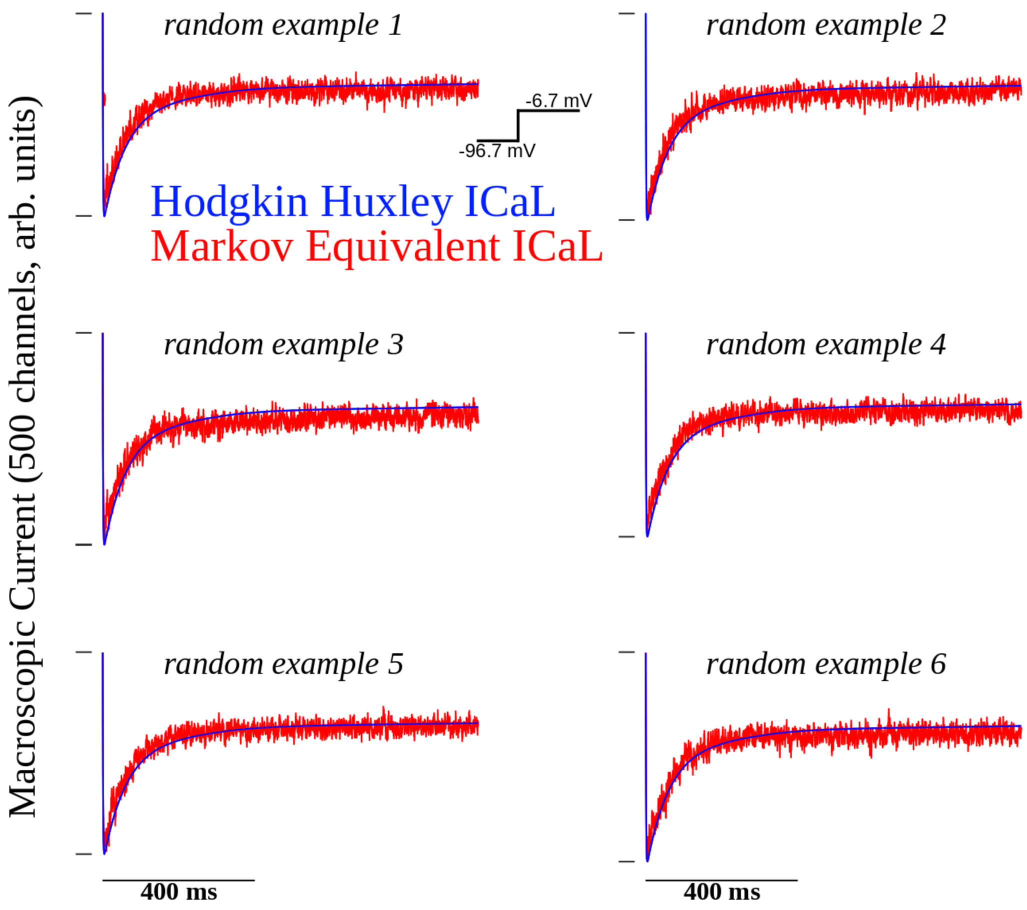

Online Figure IV. Examples of summation of an ensemble of 500 stochastically gating LTCCs. Ensemble traces in red were compared with the deterministic HH-based current (blue), illustrating that the single channel model produces the expected results in response to a voltage step to -6.7 mV.

Online Figure VII.

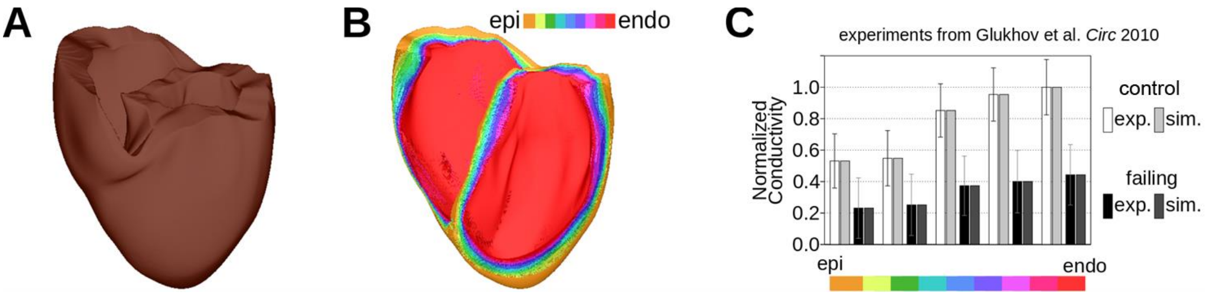

Online Figure VII. Human heart control and failing computational models. **(A)** MRI-based geometry of the human ventricular model. **(B)** Transmural cell types. **(C)** Transmural tissue conductivity values incorporated in the model, based on experimental data regarding transmural Cx43 distribution from human left ventricular wedges, as measured in Glukhov et al.[19] 2010. The values vary across the septum and free wall following the gradient pattern shown in panel **B**.

Online Figure VIII.

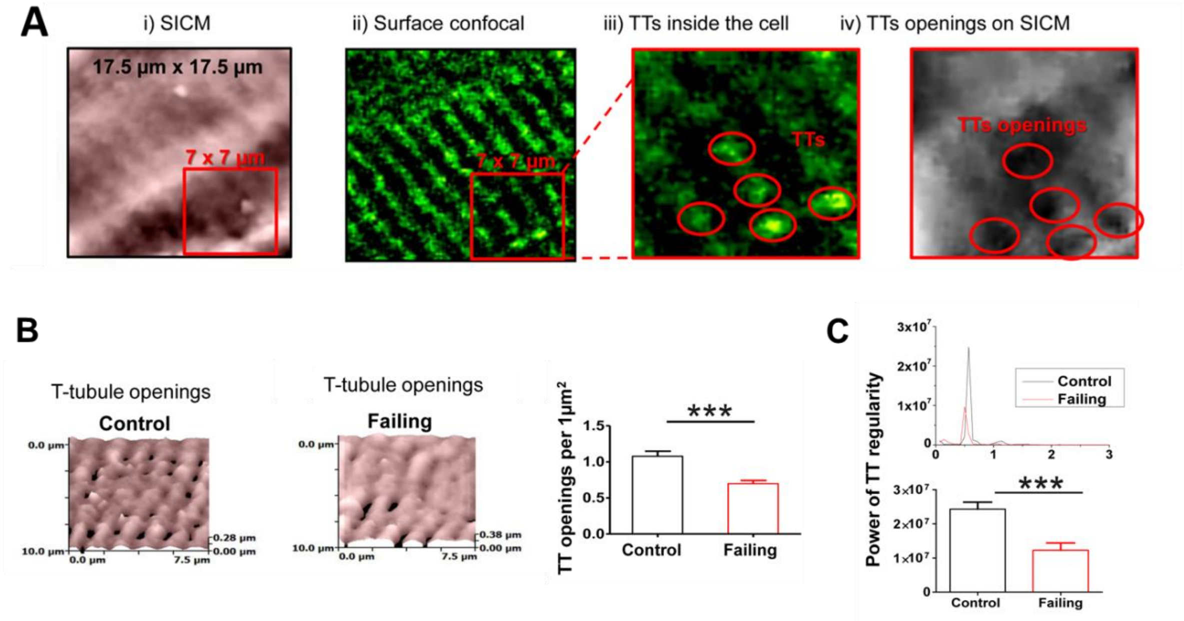

Online Figure VIII. TT openings decrease in failing cells. (A) Confocal and SICM images of the same 17.5 µm x 17.5 µm patch of membrane. The images demonstrate correlation between the two measurement modalities in both number and localization of TTs. In confocal images, di-8-ANNEPS staining (10 µM) marks the membrane. SICM images the membrane surface topology. **(B) Left,** SICM scans of a control and failing cardiomyocyte, black holes on the surface indicate TT openings. **Right,** Quantification of TT openings (control n=41 vs. failing n=44, P<0.001). **(C) Top,** power spectrum retrieved from a 2D Fourier transformation of confocal images of TT from a control and failing cardiomyocyte. **Bottom,** quantification of peak power values (in arbitrary units) at the dominant frequency (control n=11 vs. failing n=11, P<0.001).

Online Figure IX.

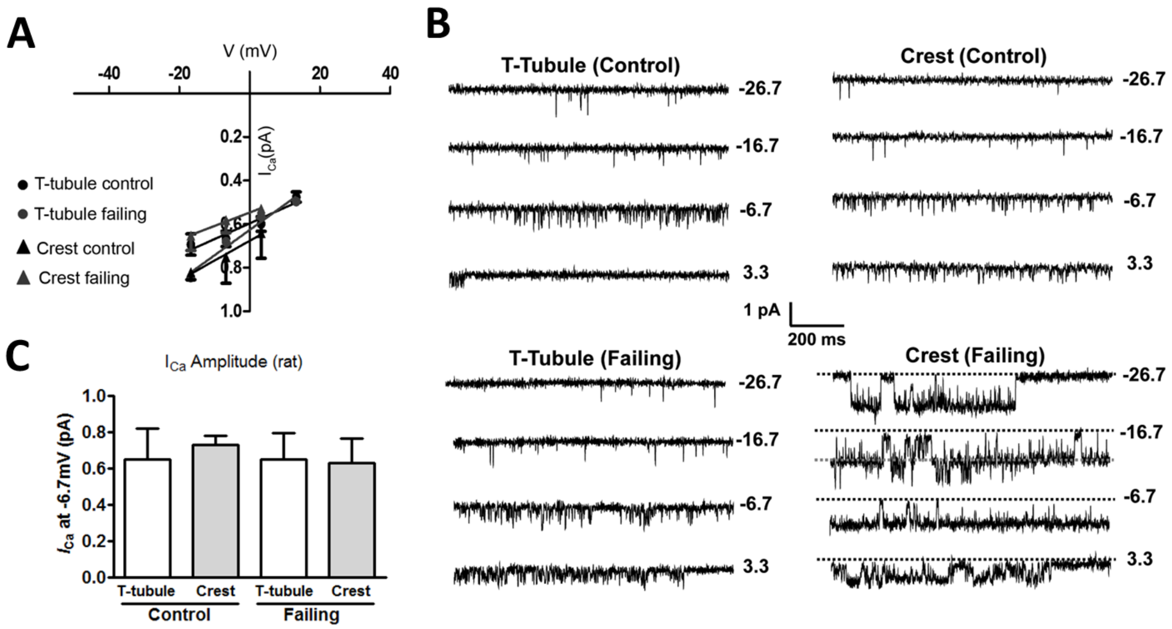

Online Figure IX. Increased LTCC activity in the crest of failing cells is not due to changes in current amplitude. (A) Voltage dependence of single LTCC currents in the TTs and in the crest of control and failing cardiomyocytes. **(B)** Representative single channel traces at indicated voltages. Downward deflections are opening of single LTCC and dotted line represents the closed level. **(C)** Single channel amplitude at -6.7 mV in control and failing rat cardiomyocytes at different locations. Amplitude was unchanged between all groups (control, TT n=19, crest n=4; failing, TT n=11, crest n=13).

Online Figure X.

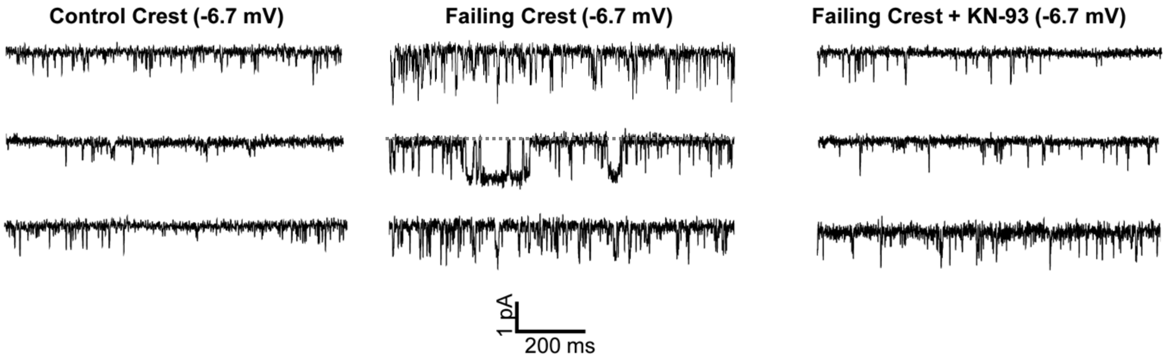

Online Figure X. KN-93 blocker reduces LTCC activity in the crest of failing cells. Representative single channel traces at -6.7 mV showing single channel activity in the crests of control, failing, and KN-93-treated failing cardiomyocytes. Dotted line represents the closed level.

Online Figure XI.

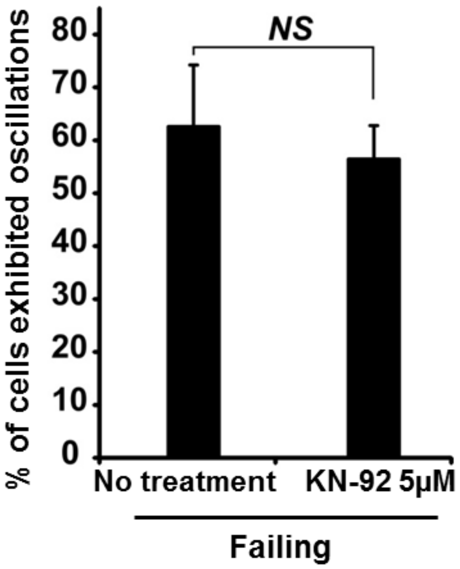

Online Figure XI. Effect of KN-92 on failing cardiomyocytes. Percentage of mapped cells that exhibited oscillations are shown for failing cells before and after treatment by KN-92 (n=2 rats for each treated group).

Online Figure XII.

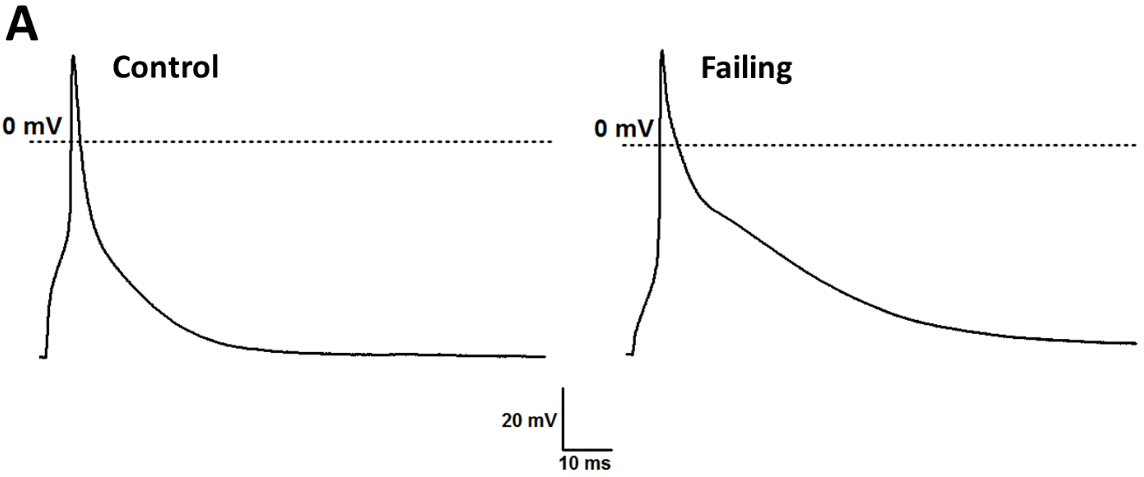

B

Group	n	Cm (pF)	RMP (mV)	APA (mV)	dV/dt_{max} (V/s)	APD_{50} (ms)	APD_{90} (ms)
**Control RAT**	12	226±21	-70.7±1.2	98.5±2.3	152±15	14.3±1.8	62.7±6.2
**Failing RAT**	12	337±23**	-67.7±0.9	97.5±2.3	162±15	20.3±2.1*	101.2±16.4*
**p-value**		**0.0056**	0.14	0.75	0.63	**0.0391**	**0.0385**

Online Figure XII. Action potential recordings and characteristics from control and failing rat cardiomyocytes. (A) Representative AP trace of a control (left) and a failing rat cardiomyocyte (right) after 5ms threshold pulse at 1Hz stimulation. **(B)** AP characteristics. Cm indicates membrane capacity. RMP indicates resting membrane potential. APA indicates action potential amplitude. APD indicates action potential duration at 50% and 90%. Values are mean±SEM.

Online Figure XIII.

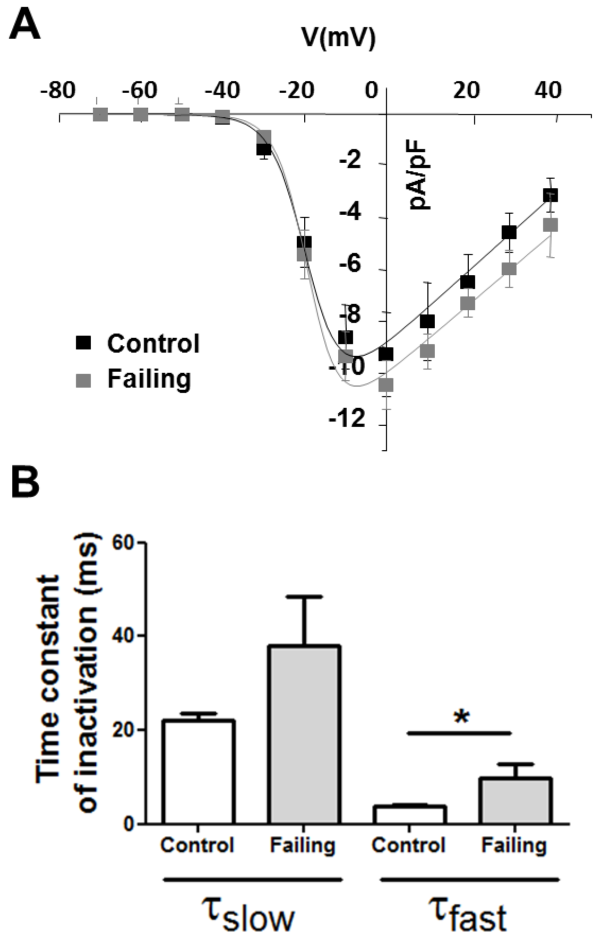

Online Figure XIII. Whole cell L-type calcium current recordings from control and failing rat cardiomyocytes. (A) Whole cell I_{CaL} current-voltage relationship of control and failing cardiomyocytes (control n=7, failing n=5). **(B)** Voltage dependence of the time constant of I_{CaL} inactivation in both control and failing cardiomyocytes from A. * indicates $p<0.05$.

Online Figure XIV.

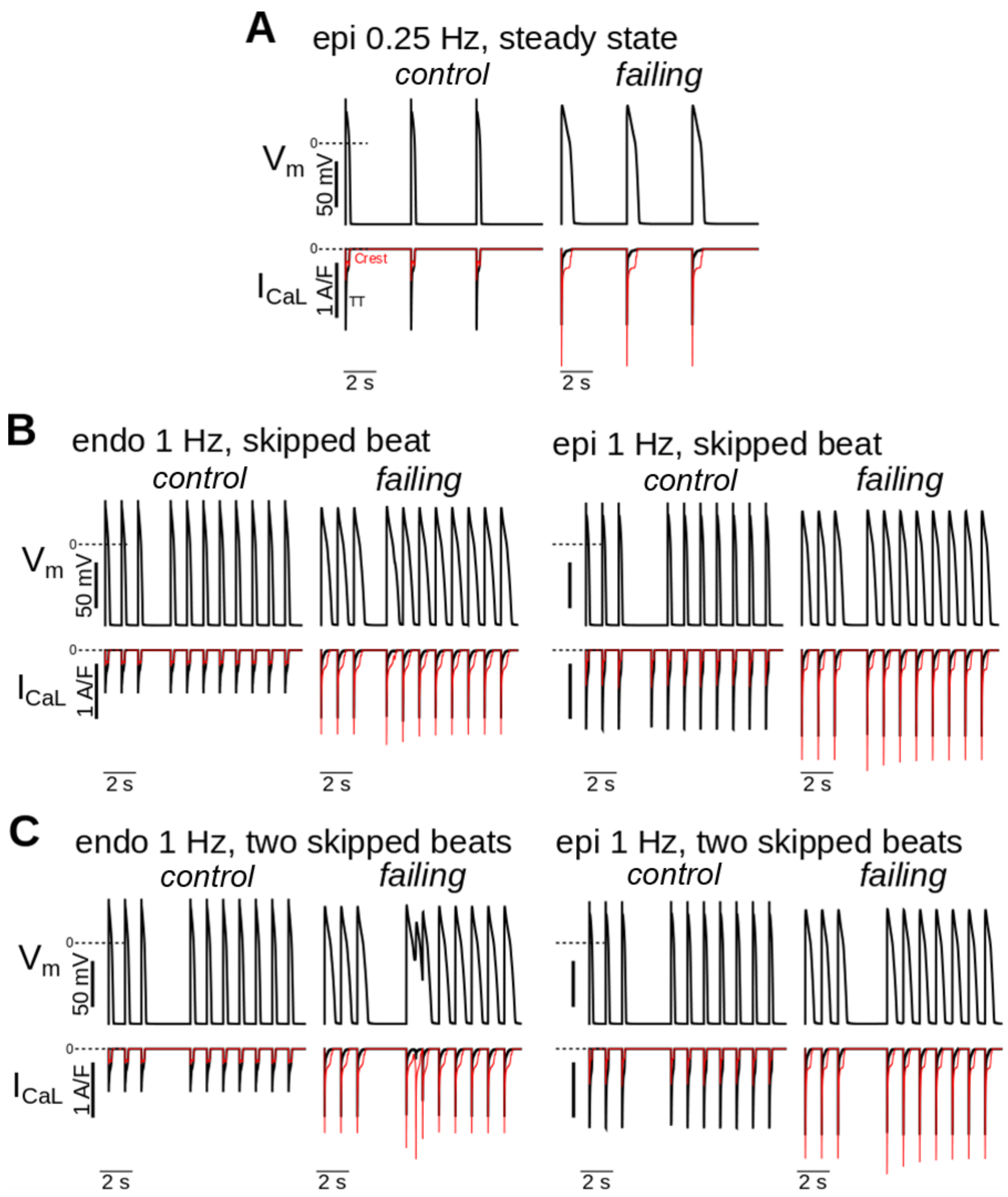

Online Figure XIV. Simulations of EADs in failing ventricular myocytes as a function of transmural cell type and pacing protocol. **(A)** Slow pacing at 0.25Hz did not result in EADs in control (left) or failing (right) human epicardial (epi) cells. **(B)** Pacing human endocardial (endo, far left is control, and second from left is failing) or epi cells (control and failing are second from right and far right, respectively) at 1Hz with a single skipped beat did result in EADs. **(C)** 1 Hz pacing with two skipped beats resulted in an EAD in failing human endo cells (second from left), but not in the other cases.

Online Tables.

Patient	Diagnosis	Age	Gender	NYHA	BNP (ng/l)	LV Post Wall Thick. Systole (mm)	LV Post Wall Thick. Diastole (mm)
**HF#1**	DCM	40	Male	IV	2839	13	9
**HF#2**	DCM	33	Male	IV	624	10	8
**HF#3**	DCM	57	Male	IV	1525	10	8
**HF#4**	DCM	56	Female	IV	1543	7	6
**HF#5**	DCM	64	Male	IV	942	6	8
**HF#6**	DCM	41	Male	IV	588	10	7

Online Table I. Heart failure patients characteristics. HF, heart failure; DCM, dilated cardiomyopathy; NYHA, New York Heart Association functional classification; BNP, B-type Natriuretic Peptide levels.

Patient	Diagnosis	Age	Gender
**NF#1**	MVR	78	Female
**NF#2**	MVR	69	Male
**NF#3**	MVR	66	Male
**NF#4**	MVR	70	Female
**NF#5**	MVR	64	Male

Online Table II. Control patients characteristics. NF, nonfailing; MVR indicates mitral valve replacement procedure.

Group	successful seal rate	Total cells sealed	Occurrence (cells that show LTCC activity)	Pipette resistance (MΩ)	
				TT	Crest
**Control RAT**	76%	143	19%	32.34±0.85	30.48±1.41
**Failing RAT**	70%	99	26%	31.93±1.4	30.02±0.75
**Control HUMAN**	84%	33	22%	30.52±1.23	25.73±2.76
**DCM HUMAN**	75%	48	31%	33.32±1.49	25.64±1.16

Online Table III. Patch-clamp single channels seals characteristics. Percentage of successful seals was calculated from the total number of seals tried after the clipping of the pipette. Overall, 7% of the pipettes were discarded after the clipping process and were not used for the analysis. Pipette resistance corresponded to an estimated inner tip diameter range of 260-425 nm for TT and 260-530 nm for Crest recordings.

Online Videos

Online Video I. Simulated failing human ventricular movie showing the formation of arrhythmogenic triggers and reentrant arrhythmia. (1Hz pacing with a single skipped beat after two pacing stimulus), after the skipped beat cells failed to repolarize and an endocardial EAD trigger formed. Note that the activation took twice as long to excite the failing ventricles when compare to control.

Online Video II. Simulated control human ventricular movie showing organize and regular response to each pacing stimulus (1Hz pacing with a single skipped beat after two pacing stimulus). The skipped beat does not produce any effect on the control ventricle.
